# Predicting Early Allograft Function After Normothermic Machine Perfusion

**DOI:** 10.1097/TP.0000000000004263

**Published:** 2022-08-29

**Authors:** Christopher J.E. Watson, Rohit Gaurav, Corrina Fear, Lisa Swift, Linda Selves, Carlo D.L. Ceresa, Sara S. Upponi, Rebecca Brais, Michael Allison, Corrie Macdonald-Wallis, Rhiannon Taylor, Andrew J. Butler

**Affiliations:** 1 Department of Surgery, University of Cambridge, Level E9, Addenbrooke’s Hospital, Cambridge, United Kingdom.; 2 The National Institute of Health Research, Cambridge Biomedical Research Centre (BRC 1215 20014), Cambridge, United Kingdom.; 3 The National Institute for Health Research Blood and Transplant Research Unit, University of Cambridge in collaboration with Newcastle University and in partnership with National Health Service Blood and Transplant, Cambridge, United Kingdom.; 4 The Roy Calne Transplant Unit, Cambridge University Hospitals NHS Foundation Trust, Cambridge, United Kingdom.; 5 Nuffield Department of Surgical Sciences, University of Oxford, Oxford, United Kingdom.; 6 Department of Radiology, Cambridge University Hospitals NHS Foundation Trust, Cambridge, United Kingdom.; 7 Department of Pathology, Cambridge University Hospitals NHS Foundation Trust, Cambridge, United Kingdom.; 8 Department of Medicine, Cambridge University Hospitals NHS Foundation Trust, Cambridge, United Kingdom.; 9 Statistics and Clinical Research, National Health Service Blood and Transplant, Bristol, United Kingdom.

## Abstract

Normothermic ex situ liver perfusion is increasingly used to assess donor livers, but there remains a paucity of evidence regarding criteria upon which to base a viability assessment or criteria predicting early allograft function.

**Methods.:**

Perfusate variables from livers undergoing normothermic ex situ liver perfusion were analyzed to see which best predicted the Model for Early Allograft Function score.

**Results.:**

One hundred fifty-four of 203 perfused livers were transplanted following our previously defined criteria. These comprised 84/123 donation after circulatory death livers and 70/80 donation after brain death livers. Multivariable analysis suggested that 2-h alanine transaminase, 2-h lactate, 11 to 29 mmol supplementary bicarbonate in the first 4 h, and peak bile pH were associated with early allograft function as defined by the Model for Early Allograft Function score. Nonanastomotic biliary strictures occurred in 11% of transplants, predominantly affected first- and second-order ducts, despite selection based on bile glucose and pH.

**Conclusions.:**

This work confirms the importance of perfusate alanine transaminase and lactate at 2-h, as well as the amount of supplementary bicarbonate required to keep the perfusate pH > 7.2, in the assessment of livers undergoing perfusion. It cautions against the use of lactate as a sole indicator of viability and also suggests a role for cholangiocyte function markers in predicting early allograft function.

## Graphical abstract

**Figure:**
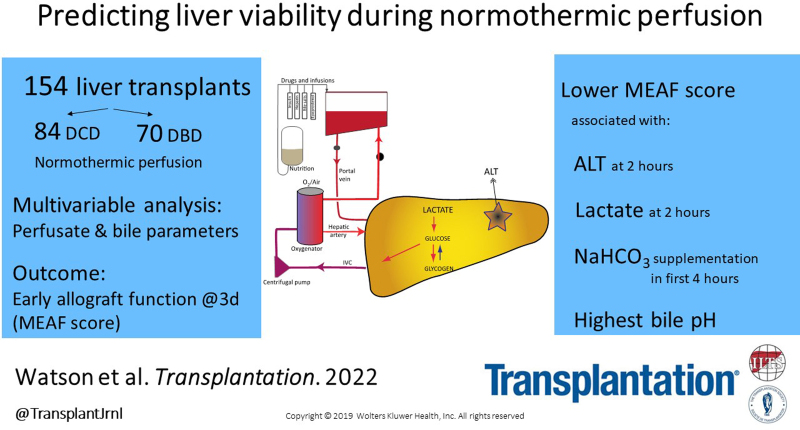

## INTRODUCTION

The last decade has seen the development of new techniques to preserve livers and improve outcomes after transplantation. These range from donor interventions‚ such as in situ normothermic regional perfusion (NRP) in donation after circulatory death (DCD) donors,^1,2^ to periods of ex situ normothermic or hypothermic perfusion starting either at the donor hospital or after arrival at the recipient center. These interventions facilitate prolonged storage, organ optimization, and/or assessment, a period variously labeled as recovery, repair, or resuscitation, although evidence for the latter two processes is lacking.^3-5^

In 2018, we published biochemical variables that were associated with successful posttransplant outcomes in livers subject to normothermic ex situ perfusion (NESLiP), based on our early experience with 47 livers, most of which had been turned down for transplantation by other centers (Table 1).^6^ Our aim was to identify parameters common to functioning livers, and in livers that went on to develop cholangiopathy, while highlighting factors present in the 1 liver that suffered primary nonfunction (PNF).

**TABLE 1.** Factors associated with a successful transplant

	Factors associated with successful transplant using XVIVO liver assist^6^	Revised parameters associated with successful transplants based on data accrued for the OrganOx metra^a^
Cholangiocyte function		
Bile chemistry		
pH	Maximum pH > 7.5	Maximum pH > 7.6
Glucose	≥10 mmol less than perfusate or, if perfusate glucose <11 mmol/L, a bile glucose ≤3 mmol/L	≥10 mmol less than perfusate or, if perfusate glucose <11 mmol/L, a bile glucose ≤2 mmol/L
Hepatocyte function		
Perfusate chemistry		
pH	Able to maintain pH > 7.2 with ≤30 mmol NaHCO_3_	Continuing requirement for bicarbonate to maintain pH > 7.2 beyond 2 h.The need for bicarbonate in the first 2 h to maintain a pH > 7.2 is no longer considered important if other parameters are satisfactory.
Glucose	Glucose falling beyond 2 h or a glucose <10 mmol/L, which, on challenge, with 2.5 g dextrose, does fall	If glucose <10 mmol/L at 30 min, a 5-g dextrose challenge should be given.The glucose rate of fall should be ≥1 mmol/L/h by 4 h.
Lactate	Peak lactate fall in first hour ≥4.4 mmol/L/kg/h	2 h lactate <2.8 mmol/L
ALT	<6000 u/L at 2 h	ALT <6000 u/L at 2 h

*^a^*The current study is too small to safely exclude the importance of all factors, but it emphasized the importance of ALT and 2-h lactate. The values of 2-h lactate and ALT quoted are the highest associated with a successful transplant in our series so far using the OrganOx metra and may not apply to other devices.

These criteria are not and were never meant as thresholds for viability but rather reflected our practice, which has become less conservative as experience has accrued. Given the dire consequences of primary nonfunction, transplanting livers at the margins should only be undertaken after careful recipient selection and after informing the recipient of the uncertainties involved.

We continue to look at bile glucose and H+ as markers of cholangiocyte function. The thresholds above do not exclude cholangiopathy but were associated with an incidence of 12% in these livers, most of which had been declined by other centers.

In the analysis reported here, good bile chemistry predicted good early allograft function.

ALT, alanine transaminase.

Other centers have published criteria to determine viability, but often on a small number of livers including many that had not been transplanted. The first proposal was based on 12 nontransplanted livers, suggested a bile production threshold of 30 g at 6 h (~20 mL at 4 h)‚ and discriminated viable from nonviable livers.^7^ Subsequently Mergental et al published criteria based on 6 livers, 5 of which were transplanted.^8^ They assessed livers within 3 h, at which point viability was indicated by a lactate <2.5 mmol/L or the production of bile, together with 2 of a perfusate pH >7.3, arterial flow >150 mL/min, and portal flow >500 mL/min. Subsequently, they revised their criteria extending the assessment to 4 h, keeping lactate as the key determinant but adding a need for glucose metabolism into their list of optional requirements.^9^ These criteria did not include any bile chemistry, and DCD liver recipients following these criteria experienced a 30% incidence of nonanastomotic biliary strictures (NAS) at 12 mo.^9^

Other suggested viability criteria have been reviewed elsewhere^10^ and include criteria relating to bile production, lactate and glucose metabolism, pH regulation, and release of transaminases. Biliary pH and glucose metabolism have been included in most criteria since publication of our original observations.^11^

To properly define viability criteria‚ it is necessary to have perfusions at both extremes of good and bad, including livers suffering PNF Model for Early Allograft Function. In our series to date, which includes 192 transplants since 2014, mostly of higher-risk donor livers, we have had 2 livers suffering PNF.^11^ Because this is insufficient to determine viability, we have instead chosen to correlate biochemical parameters during perfusion with early graft function. There are several metrics available, including the Olthoff criteria, the Model for Early Allograft Function (MEAF) score, the Liver Graft Assessment Following Transplantation (L-Graft) score, and the Early Allograft Failure Simplified Estimation score.^12-15^ All have been defined to estimate the likelihood of early graft failure in livers subject to preservation on ice. Because we do not routinely measure aspartate transaminase (AST) posttransplant, preferring alanine transaminase (ALT), the L-Graft and Early Allograft Failure Simplified Estimation scores could not be calculated; the Olthoff score was also discounted because of its categorical nature‚ leaving the MEAF score against which perfusion variables were assessed.

In this study, we sought to determine which perfusion factors, including those suggested by ourselves and others,^7,16^ were associated with early allograft function as determined by the MEAF score. We examined whether bile chemistry, including bile:perfusate glucose concentration ratio, predicted NAS.^17^

## MATERIALS AND METHODS

### Indication for Liver Perfusion

Livers underwent NESLiP when there was concern over viability, logistical reasons preventing immediate transplantation, or recipient considerations such as anticipation of prolonged surgery or physiological instability for which NESLiP was used to minimize reperfusion hemodynamic disturbance. All but 1 DCD liver in the study period either underwent in situ NRP or NESLiP before implantation.

### Liver Perfusion

The OrganOx metra device was used for NESLiP in all cases. The perfusate comprised group-compatible red cells suspended in either succinylated gelatin (Gelofusine, BBraun, n = 54) or 4.5% Human Albumin Solution (n = 149), to which boluses of heparin, amino acids, magnesium, calcium, meropenem, and fluconazole were added. Gas flow to the oxygenator is regulated automatically based on real-time analysis of the perfusate gas concentrations by an inbuilt Terumo module. Throughout perfusion, livers received infusions of heparin, sodium taurocholate, insulin, and epoprostenol, with a supplementary 20% dextrose infusion when perfusate glucose was <10 mmol/L.

### Monitoring

Perfusate and bile samples were assayed using either a Cobas b221 (Roche Diagnostics, Indianapolis, IN) or a RAPIDPoint 500e (Siemens) point of care blood gas analyzer, the latter being used for the last 55 cases. A pH >8.0 was above the range of the Cobas analyzer and >7.8 was above the range of the RAPID point analyzer; pH values >7.8 were, therefore, defaulted to 7.8. ALT concentrations were measured at 60 and 120 min using a Piccolo Xpress point of care device (Abbott, UK); values over 2000 u/L were diluted 1 in 3 in saline; diluted values >6000 u/L were assayed in the hospital’s biochemistry department.

### Markers Predicting Early Allograft Function

In addition to factors previously thought to predict early allograft function (Table 1), we also recorded lactate concentration, volume of bile produced, bile glucose, and pH hourly to 4 h. Donor graft “quality” was assessed using the US Donor Risk Index^18,19^ and United Kingdom Donor Liver Index.^20^ The absence of bile production in a DCD liver was considered a contraindication to its use because cholangiocyte viability could not be assessed; this was not a contraindication for donation after brain death (DBD) livers.

Posttransplant allograft function was evaluated by the MEAF score, which ranges from 0 to 10‚ with higher values indicating poorer early allograft function.^13^ Magnetic resonance cholangiopancreatography was done when clinically indicated. Anastomotic strictures were defined as those requiring surgery or stenting, and NAS was defined as strictures involving first-order or higher ducts.

### Ethical Approval

The study was approved by the Human Biology Research Ethics Committee of the University of Cambridge (HBREC.2020.23).

### Statistical Analysis

A detailed description of the statistical methods is given in Supplemental Statistical Methods (**SDC**, http://links.lww.com/TP/C550). Essentially, a descriptive analysis of variables was performed, and comparisons were made between transplanted and nontransplanted livers. Linear regression models with MEAF scores as outcomes, regressed on the perfusion variables, were fitted after first normalizing the outcome variables of interest. The best fitting transformation for the MEAF score was a square root transformation. Univariable linear regression models were fitted with each of the perfusion variables in turn as the exposure variable, the variables being log-transformed in case of skew distribution. A multivariable model for MEAF was fitted with variables showing the strongest association.

## RESULTS

Between May 1, 2017, and February 1, 2022, 203 livers underwent NESLiP at our center (80 DBD, 123 DCD); in 27 DCDs, NESLiP followed NRP, 18 of which were transplanted. One more liver was perfused as part of a liver and lung transplant and has not been included in the analysis. Weights were available for 159 (78%) livers and ranged from 614 to 2800 g (median 1560 g).

Table 2 details donors, recipients, and perfusion characteristics by donor type. Of the 203 livers undergoing NESLiP, 84/123 (68%) DCD and 70/80 (88%) DBD livers were transplanted; the reason for discarding a liver is given in **Table S1, SDC**, http://links.lww.com/TP/C550. One patient developed PNF, and 17 of the 147 (12%) recipients with 7 d data developed early allograft dysfunction by the criteria of Olthoff et al.^12^ One-year actuarial death-censored graft survival was 95.4%, uncensored graft survival 91.2%, and patient survival 94.0% (**Figure S1, SDC**, http://links.lww.com/TP/C550).

**TABLE 2.** Recipient and donor factors and perfusion data

Transplanted or not	Livers donated after circulatory death		Livers donated after brain death	
	Transplanted	Not transplanted	Transplanted	Not transplanted
Number of livers	84	39	70	10
Recipient age, y	56 (48, 61)		56 (47, 64)	
Recipient MELDNa	15.3 (12.4, 19.5)		18.4 (13.8, 24.3)	
UKELD	53 (50, 57)		54 (52, 60)	
Donor age, y	48 (30, 57)	50 (36, 59)	50 (31, 59)	55 (51, 63)
Cold ischemic time, min	395 (338, 444)	432 (357, 489)	462 (362, 584)	495 (401, 591)
NESLiP duration, min	507 (434, 648)	358 (262, 434)	424 (293, 559)	352 (211, 525)
US Donor Risk Index^19^	2.3 (2.0, 2.6)	2.3 (1.9, 3.0)	1.6 (1.5, 2.0)	2.0 (1.9, 2.3)
UK Donor Liver Index^20^	1.8 (1.6, 2.2)	2.0 (1.7, 2.2)	1.0 (0.9, 1.2)	1.2 (1.0, 1.3)
UK DCD risk score,^21^ n (%)	49 (58%) “low risk” 27 (32%) “high risk” 8 (10%) “futile”			
Liver weight, g	1576 (1348, 1803)	1646 (1360, 1965)	1521 (1300, 1798)	1760 (1578, 2042)
Early allograft dysfunction^12^	10/80 = 12.5%		8/70 = 11.4%	
Model for Early Allograft Function Score	3.7 (2.6, 5.5)		4.0 (2.8, 5.4)	
ALT at 1 h	1731 (1003, 2748)	2682 (1294, 6937)	957 (457, 1525)	8207 (4758, 14368)
ALT at 2 h	1800 (1116, 3042)	3654 (2145, 6283)	1033 (533, 1741)	6449 (4171, 11408)
Additional bicarbonate in first 2 h, mmol	20 (10, 30)	30 (20, 40)	20 (10, 25)	40 (20, 51)
Additional bicarbonate between 2 and 4 h, mmol	0 (0, 0)	0 (0, 0)	0 (0, 0)	0 (0, 0)
Bile volume at 2 h, ml	5.4 (0, 10.4)	3.6 (0, 7.6)	7.0 (0.5, 13.3)	0.9 (0, 4.4)
Bile volume at 3 h, ml	12.4 (4.2, 19.5)	7.5 (1.5 16.7)	17.0 (5.9, 26.0)	2.0 (0, 13.1)
Bile volume at 4 h, ml	18 (8.7, 29.4)	12.1 (6.6, 21.5)	24.4 (6.7, 32.3)	4.2 (0.5, 19.4)
Lactate peak rate of fall, mmol/L/h	16.8 (12.0, 20.2)	12.0 (8.2,16.7)	20.2 (13.5, 24.9)	12.4 (5.8, 15.7)
Lactate at 1 h, mmol/L	1.4 (0.3, 4.0)	5.4 (1.2, 8.3)	0.9 (0.4, 1.9)	6 (1.5, 8.3)
Lactate at 2 h, mmol/L	0.5 (0.2, 1.1)	0.8 (0.5, 1.8)	0.9 (0.4, 1.4)	1.6 (0.9, 3.2)
Lactate at 3 h, mmol/L	0.5 (0.3, 1.3)	0.8 (0.4, 2.3)	0.8 (0.4, 1.4)	1.9 (1.4, 2.9)
Lactate at 4 h, mmol/L	0.6 (0.3, 1.3)	0.8 (0.4, 2.3)	0.7 (0.3, 1.2)	1.7 (1.2, 2.2)
Glucose rate of fall, mmol/L/h	3.6 (2.4, 5.4)	2.8 (1.2, 3.9)	3.7 (2.6, 4.7)	2.0 (-1.6, 3.8)

Data are presented as median (interquartile range).

For the US Donor Risk Index (DRI), the correct DRI equation quoted in Shaubel et al^19^ was used, and cold ischemia time from organ recovery to start of NESLiP was used in the calculation, not total preservation time.

ALT, alanine transaminase; DCD, donation after circulatory death; MELDNa, Model for End-stage Liver Disease sodium^22^; NESLiP, normothermic ex situ liver perfusion; UKELD, United Kingdom Model for End-stage Liver Disease.

MEAF scores were not available for 6 patients; 4 died perioperatively‚ and 2 suffered early graft losses (**Table S2, SDC**, http://links.lww.com/TP/C550). Bile volume was not recorded for 19 of the first 20 perfusions.

### Analysis of Variables Previously Identified to be Important

Table 1 lists the factors previously identified as being associated with a successful transplant, and **Table S3 (SDC**, http://links.lww.com/TP/C550) identifies how many of the transplanted and nontransplanted livers met those criteria. Eighty-nine (58%) transplanted livers met all 6 criteria, with a further 28 (18%) meeting all but the weight-adjusted peak fall in lactate concentration because they had not been weighed; their peak rate of lactate fall was between 5.1 and 31.3 mmol/L/h (median 15.0 mmol/L/h).

More livers from DCD than DBD donors required >30 ml sodium bicarbonate in the first 4 h to achieve perfusate pH > 7.2 (DCD 36/123 [29%] versus DBD 16/80 [20%], *P* = 0.1921), and 24 livers failing this criterion were successfully transplanted. Only 5 (3%) of the transplanted livers needed supplementation after 2 h (1 DBD and 4 DCD), and 5 (10%) of the nontransplanted livers needed supplementation after 2 h.

**FIGURE 1.**
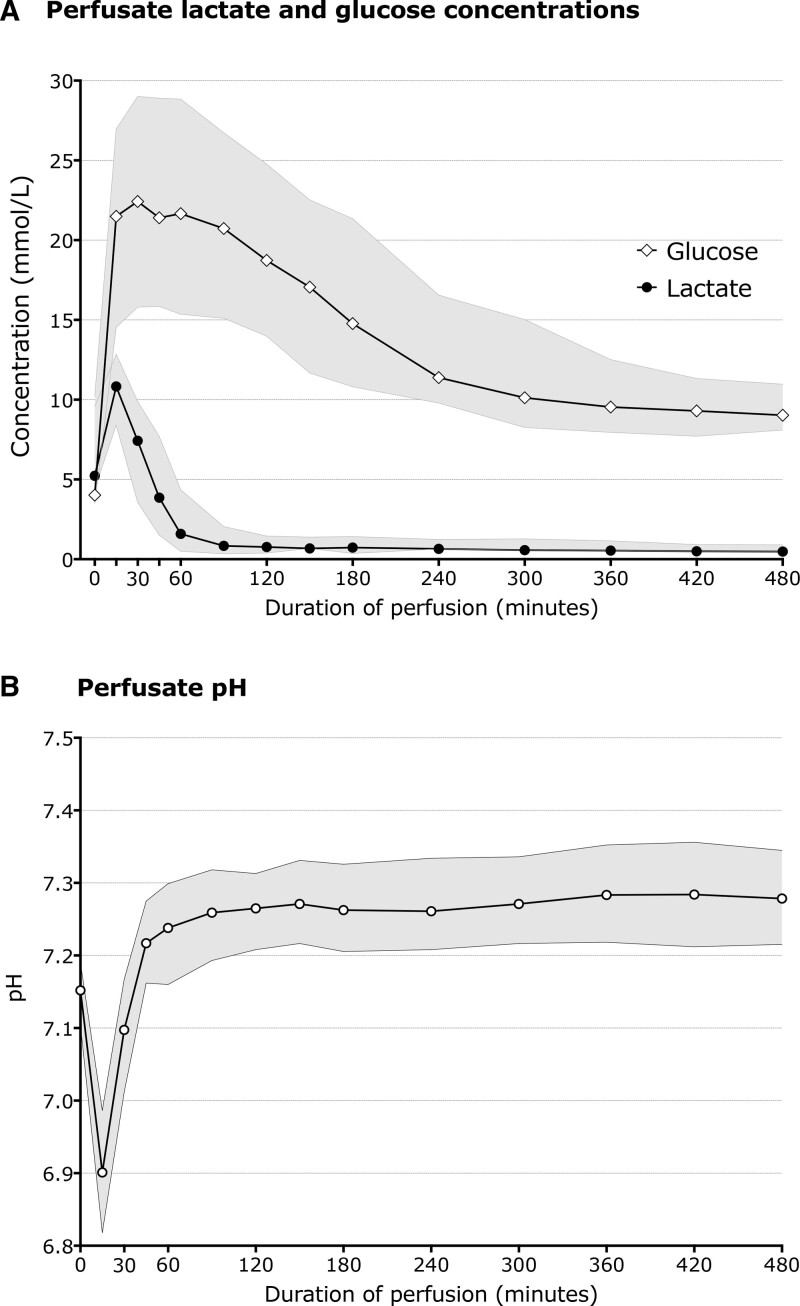
Graphs showing median and interquartile ranges of key perfusion variables over time in the livers in this series. Time zero on all the graphs is the parameter concentration in the perfusate before the liver is added. A, Rate of change of perfusate lactate and glucose; B, Change in pH over time, with a notable fall when the liver is placed on.

Figure 1 shows the change in median perfusate lactate, glucose, and pH during perfusion. The median 4-h lactate in transplanted livers was 0.6 mmol/L (interquartile range [IQR], 0.3–1.2), with 2 having 4-h lactate over 2.6 mmol/L; the maximum was 4.5 mmol/L, and that liver had a MEAF score of 9.2, in contrast to the next highest 4-h lactate of 3.8 mmol/L‚ which translated to a MEAF of 3.8. Of the 142 transplanted livers still undergoing perfusion at 4 h, 63 (44%) had produced <20 ml bile, including 10 that produced no bile. Of livers producing <20 ml bile, the median MEAF was 4.1 (IQR‚ 2.8–5.7) compared with 3.6 (IQR‚ 2.2–4.8) for those producing ≥20 ml bile. Of the 48 nontransplanted livers that were perfused for at least 4 h, 73% had produced <20 ml.

Distribution of Perfusion Parameters and Correlation With MEAF

Livers declined for transplantation had significantly different perfusion parameters compared with transplanted livers (**Figures S2–S8, Table S4, SDC**, http://links.lww.com/TP/C550. There were significant positive correlations of the square-root transformed MEAF score with ALT at 1 and 2 h; additional bicarbonate; lactate at 1, 2, and 3 h; a lactate <1 mmol/L at 1, 2, 3, and 4 h; bile volume at 2, 3, and 4 h; and the lowest bile glucose during perfusion (**Figure S9, Table S5, SDC**, http://links.lww.com/TP/C550). Of the nonperfusion variables, donor age, liver weight, the use of NRP, and lower posttransplant ratios of baseline to peak creatinine levels on days 1–7 were associated (**Table S6, SDC**, http://links.lww.com/TP/C550).

### Univariable Analysis

A complete case dataset was selected that consisted of livers for which there was complete data on each candidate variable identified above. This included 108 of the total 149 transplanted livers for which a MEAF score was available (70%); these are compared in **Tables S7 and S8 (SDC**, http://links.lww.com/TP/C550). Upon univariable analysis, perfusate concentrations of ALT, lactate, additional bicarbonate requirement to maintain pH, bile production, and lowest bile glucose concentration were strongly associated with the square root-transformed MEAF score (**Figure S9, Table S9, SDC**, http://links.lww.com/TP/C550). **Table S10 (SDC**, http://links.lww.com/TP/C550) shows the mean difference in the square root of MEAF associated with each of the variables in the univariable analysis.

### Multivariable Analysis

Candidate variables that showed the strongest univariable associations with the MEAF score were selected for consideration to be included in a multivariable model for the prediction of the MEAF. Multivariable Model A contained 6 of the candidate perfusate variables: log_e_ ALT at 2 h, additional bicarbonate in the first 2 and 4 h, lactate at 2 h, lactate <1 mmol/L at 1 h, and peak bile pH (Table 1). Because lactate <1 mmol/L at 1 h was highly correlated with lactate at 2 h, and bicarbonate in the first 2 and 4 h was highly correlated, only the 2-h lactate and bicarbonate supplementation in the first 4 h were retained in the model to produce Model B. Model B, therefore, included ALT and lactate at 2 h, bicarbonate supplementation in the first 4 h, and the peak bile pH. None of the other candidate variables showed statistical evidence of association with the MEAF score after adjustment for these 4 variables (all *P* > 0.1). There was a positive association of log_e_ 2-h ALT with the square root of MEAF in Model B and evidence of a positive association between bicarbonate requirement, lactate at 2 h, and the peak bile pH (Table 3).

**TABLE 3.** Mean difference in the square-root of the MEAF score associated with a unit increase in each of the machine perfusion variables in the final multivariable linear regression model

Variable		Multivariable association with square root of MEAF			
		Mean difference (95% CI)	Overall *P*	R squared	Adjusted R squared
**Model A. Machine perfusion factors significant in multivariable model**					
ALT at 2 h	Logged	0.30 (0.19 to 0.42)	<0.001	0.372	0.328
Additional bicarbonate in first 4 h, mmol	0–10	0	0.013		
	11–29	0.59 (0.13 to 1.05)			
	≥30	−0.12 (−0.35 to 0.12)			
Lactate at 2 h	Linear	0.19 (0.07 to 0.31)	0.003		
Bile peak pH	Linear	1.06 (0.003 to 2.11)	0.049		
Lactate at 1 h <1 mmol/L	No	0	0.035		
	Yes	0.24 (0.02 to 0.46)			
Additional bicarbonate in first 2 h, mmol	0–10	0	0.073		
	11–29	−0.42 (−0.87 to 0.04)			
**Model B. Machine perfusion factors significant in multivariable model (excluding lactate <1 mmol/L at 1 h and additional bicarbonate 0–2 h**)^a^					
ALT at 2 h	Logged	0.26 (0.16 to 0.37)	<0.001	0.330	0.297
Additional bicarbonate in first 4 h, mmol	0–10	0	0.005		
	11–29	0.22 (−0.009 to 0.45)			
	≥30	−0.11 (−0.35 to 0.13)			
Lactate at 2 h	Linear	0.12 (0.018 to 0.24)	0.023		
Bile peak pH	Linear	0.91 (−0.15 to 1.97)	0.092		

*^a^* Lactate <1 at 1 h and 2-h lactate closely correlated, and therefore lactate at 2 h was selected. Similarly, bicarbonate supplementation between 0 and 2 h, was closely correlated with supplementation between 0 and 4 h, so 0–4 was selected.

ALT, alanine transaminase; CI, confidence interval; MEAF, Model for Early Allograft Function.

The R-squared value of the final multivariable regression model was 0.33, and the adjusted R-squared value (which takes into account the number of explanatory variables in the model) was 0.297. This suggests that around 30% of the variance in the square root of the MEAF is explained by the machine perfusion variables studied.

### Cholangiocyte Parameters and Development of Nonanastomotic Biliary Strictures

Cholangiocyte criteria were either not met (5 DBD, 10 DCD) or no bile was produced (2 DBD, 1 NRP-DCD) in 17 of the 154 (11%) transplanted livers; glucose criteria in 13 (4 DBD, 9 DCD) and pH ≤ 7.5 in 2 (1 DCD and 1 DBD). One DBD liver was transplanted‚ which failed both pH and glucose criteria without biliary complications.

Seventeen (11%) livers (11 DCD, 6 DBD) developed NAS, 9 (7 DCD, 2 DBD) of which also developed anastomotic strictures requiring intervention. All 17 had a peak bile pH >7.5, and in 13 it was >7.7. Six had a maximum perfusate–glucose difference of under 10 mmol/L, 2 of which had minimum bile glucose over 3 mmol/L. Of those livers not developing NAS, 1 DBD liver had a bile pH <7.5; it also failed glucose criteria. Twenty livers had minimum perfusate glucose >3 mmol/L, 11 of which also had a maximum perfusate–bile glucose difference <10 mmol/L, and were transplanted based on a high bile pH.

Of the 16 livers developing NAS‚ which had produced bile by 4 h, the bile:perfusate glucose ratio was <0.4 in 15 (94%) and 0.76 in the remaining one. The ratio was >0.67 in 7 of 113 livers for which the ratio could be measured, with only 1 developing cholangiopathy.

## DISCUSSION

This article has assessed how perfusion variables relate to early allograft function posttransplant, as determined by the MEAF score. We have shown in a multivariable analysis of perfusion parameters that the MEAF score is associated with perfusate ALT at 2 h, perfusate lactate at 2 h, a need for 11-29 mmol/L supplementary bicarbonate to achieve a pH >7.2, and peak bile pH. Several factors need to be taken into consideration in interpreting these data.

The MEAF score reflects hepatocyte damage and function in the first 3 d posttransplant and is based on ALT, bilirubin, and international normalized ratio; it has been shown to predict early graft survival in non-NESLiP livers.^13,23,24^ It is possible that MEAF does not predict survival in NESLiP livers, and this may account for some of the loss of predictive value of the variables studied. We did explore the utility of the L-Graft score, substituting ALT for AST, but found no correlation between factors thought to predict early allograft function possibly because the substitution of AST may have reduced its predictive value. The Innsbruck group has also failed to demonstrate a correlation between the factors they thought predicted early allograft function and the L-Graft score.^25^ One reason may be the reliance of such scores on the posttransplant ALT, measuring ALT that is washed out of the transplanted liver. Because some ALT washout occurs during NESLiP, posttransplant washout is likely to be less representative of the magnitude of reperfusion injury. Another explanation might be that these scores are designed to predict graft survival and not early posttransplant function. Further work needs to be done on NESLiP-treated livers to determine a satisfactory metric of early function, and we would encourage the development of national and international registries to address this. An alternative approach would be a formal assessment of hepatocellular function in the postoperative period by administering diagnostic reagents to the recipient.

The choice of measured parameters has been dictated in part by the ease of near-patient measurement of lactate, glucose, and pH and in part because they reflect activity across the hepatic lobule: lactate metabolism is oxygen dependent and occurs predominantly in zone 1, whereas glucose incorporation into glycogen begins in zone 3, with the regulation of pH depending both on zone 1 (glutamine synthesis) and zone 3 (glutamate synthesis).^26,27^ ALT, the most strongly associated variable, is an enzyme released following hepatocellular damage and is not normally a measure of hepatocyte function; in NESLiP‚ it probably acts as a surrogate for functional capacity, reflecting the mass of damaged hepatocytes. It was preferred to AST in our studies because of the presence of the latter in red cells, which may be damaged on the artificial circuit during NESLiP. Weissenbacher et al, on a smaller population of mostly DBD livers, also showed that perfusate transaminase levels correlated with early allograft function.^25^ It is noteworthy that flavin mononucleotide, released from damaged mitochondria, has been proposed as an indicator of viability in hypothermic oxygenated liver perfusion.^28^

Of the other variables associated with MEAF, lactate is a commonly used marker of liver function and is promoted as a definitive indicator of viability by some. Given the small volume of the NESLiP circuit (median‚ 1.3 L), lactate clearance may occur despite extensive hepatocyte damage, especially of zone 3 hepatocytes‚ which are the first to be lost in ischemia, such as during the withdrawal period in a DCD donor. Raised lactate levels are thus likely to represent either significant hepatocyte dysfunction, or simultaneous generation of lactate, for example, from liver segments supplied by a missed aberrant hepatic artery or parenchyma compressed by the weight of the liver (cradle compression^29^). Contrary to the Birmingham criteria, the 4-h lactate did not emerge as a significant predictor of function in this analysis.

Analysis of variables adjusted for liver weight might be more revealing since it is likely that livers at the extremes of weight (0.6 to 2.8 kg in this study) have different thresholds for some variables. In light of the smaller proportion of livers that had been weighed preperfusion, we have not been able to present a liver weight-adjusted analysis.

The role of parameters reflecting cholangiocyte function, particularly bile pH, reflects ischemia-reperfusion injury affecting these cells, mirroring its effects on hepatocytes. This is the first time that markers of cholangiocyte function have been associated with early allograft function. What is disappointing, but understandable, is the fact that the markers of cholangiocyte function were not able to fully predict cholangiopathy. The addition of bicarbonate and extraction of glucose from bile takes place in the cholangiocytes lining the small intrahepatic canaliculi; very many of these would have to be affected to have major changes in bile chemistry. Additionally, many of the cases presented with strictures of first- and second-order ducts, beyond where the bile chemistry is changed. A measure of viscosity, reflecting the mucus production by peribiliary glands, might be a better marker of function of the cells lining these major ducts. Alternatively, cholangioscopy may be of value, although our initial experience suggests correlating cholangioscopic appearance with subsequent strictures is not straightforward. Additionally, many of the unused DCD livers in this series were not transplanted based on adverse biliary parameters, most of which were shown subsequently to have widespread areas of stromal necrosis—if these had been transplanted the cholangiocyte variables may have been more predictive. What our analysis did show was the lack of utility of the 0.67 bile:perfusate glucose ratio cut-off in discriminating ducts that would go on to exhibit cholangiopathy, contrary to the proposal of Matton et al.^17^

A significant drawback of using posttransplant scores as a read-out of early function is that nontransplanted livers are ignored, which in this series represented 24% of perfused livers. It is likely that the discarded livers, had they been transplanted, would have produced poorer MEAF scores than those that were actually selected for transplantation, and thus‚ their exclusion could have introduced selection bias into our analysis. Also, the distributions of perfusion variables in the transplanted livers differed from those in the discarded livers, although there was considerable overlap. Associations between perfusion variables and functional scores (we were not able to calculate scores for discarded livers) may be different in the range of values observed in the discarded livers from those reported here for transplanted livers, where this end of the distribution is less well-represented. To better define viability, it is necessary to have posttransplant data on these extreme livers and in particular livers that failed to function after transplantation and those that worked. To that end, it would be helpful if there was a registry of all cases of PNF and initial poor function following machine perfusion to educate users.

We have had 2 livers suffer PNF in the 192 NESLiP transplants we have done to date, 1 in this series and 1 before while using the Liver Assist. Both had an ALT >6000 u/L at 2h but low lactates at 2 h (1.4 and 1.7 mmol/L). One required 30 and the other 35 mmol bicarbonate in the first 4 h, 1 made 4 mL bile with no bile pH recorded but bile glucose <1 mmol/L, and the other made 11.3 mL bile in the first 4 h with a peak pH > 7.8 and peak bile:perfusate difference of 13.7 mmol/L. Both were DCD livers. The unique identifying characteristic of these PNF livers was initial glucose <10 mmol/L (180 mg/dL) at 15 and 30 min, which has only been observed in 9 of the 152 liver perfusions livers in the current series. The 7 non-PNF livers responded to a 3-g dextrose challenge with a rapid fall in glucose; this was not the case in the most recent PNF, for which the glucose fell 2 mmol/L over 5 h following an initial rise. The glucose challenge was instituted in response to our first PNF. In light of this, it is notable that the rate of fall of glucose was not associated with the outcome in our multivariable analysis. The infrequent occurrence of such poor outcomes and the prior selection of favorable livers to transplant probably explain this. The observation that a moderate amount of supplementary bicarbonate is associated with MEAF, as opposed to a large amount or none, is difficult to explain but may relate to selection of livers better able to maintain pH.

In this analysis, and in those by other authors,^10^ we have interpreted the speed of recovery from ischemia as a surrogate for viability, using endpoints early in the period of perfusion. This may not be the case, and it may be that the liver should undergo more prolonged perfusion to allow for fuller recovery from ischemia and reperfusion and for regeneration of lost hepatocytes before a final decision regarding viability is made. A method of interrogating liver function regularly while being perfused would be necessary to evaluate this fully.

Although it is the largest analysis to date, this study remains limited by its small size and the inclusion of only transplanted livers. We believe that it is unlikely that any analysis of machine-perfused livers‚ which have already been selected as suitable for transplantation, is likely to be able to define viability criteria in the absence of a large cohort of grafts that failed to function. The analysis we report reinforces our a priori prejudices and indicates which perfusion variables may be the strongest predictors of early allograft function, namely 2-h ALT, 2-h lactate, bile pH, and requirement for supplementary bicarbonate. We believe that the revised parameters described in Table 1 will be associated with livers that would have a satisfactory early function and give confidence to newcomers in the field, while we acknowledge that there is a gray area outside these values for which transplantation might be successful.

## Supplementary Material


