# Supplementary material for: Predicting Early Allograft Function After Normothermic Machine Perfusion
Source: Transplantation. 2022 Aug 29;106(12):2391–8. doi: 10.1097/TP.0000000000004263 (PMC9698137; doi:10.1097/TP.0000000000004263)
Supplement: Supplementary file 1 [file tp-106-2391-s001.pdf]

Predicting early allograft function after normothermic machine perfusion: Supplementary digital content

Contents

SDC: Statistical methods 2

Table S1: Reasons for discarding 49 livers that underwent NESLiP 4

Table S2. Cause of death or graft loss and MEAF at transplant 5

Table S3. Number of livers perfused and transplanted, or not transplanted, with variables previously reported by us to be associated with a successful transplant 6

Table S4 Distributions of liver donor and perfusion parameters in transplanted livers (n=154) and non-transplanted livers (n=49) 8

Table S5. Spearman correlation coefficients of machine perfusion variables versus the MEAF Score 10

Table S6. Mean difference in square-root-transformed MEAF score associated with the non-perfusion related variables included in the dataset in univariable linear regression models 11

Table S7. Distributions of candidate machine perfusion variables and the MEAF score in the full dataset (n= 150) and for livers with complete data on all candidate variables as well as the MEAF (n = 108) 12

Table S8. Distributions of other variables in full and complete candidate variable cohort 13

Table S9. Mean difference in square-root transformed MEAF score associated with each of the machine perfusion variables in univariable linear regression models 14

Table S10. Mean difference in the square-root of the MEAF score associated with a unit increase in each of the machine perfusion variables in univariable linear regression model 16

Figure S1. Graft, patient and transplant survival 18

Figure S2a. Frequency distribution of the model for early allograft function (MEAF) scores for transplanted livers 19

Figure S2b. Frequency distribution of the square root transformed Model for Early Allograft Function (MEAF) scores for transplanted livers 19

Figure S2a: Frequency distribution of 2-hour perfusate ALT concentrations in transplanted and non-transplanted livers 20

Figure S2b: Frequency distribution of the natural logarithm of the 2-hour perfusate ALT concentrations in transplanted and non-transplanted livers 20

Figure S3. Frequency distribution of the 2-hour perfusate lactate concentration (mmol/L) 21

Figure S4. Frequency distribution of the number of millimoles of sodium bicarbonate supplementation required in the first 4 hours of perfusion to keep the perfusate pH >7.2, comparing transplanted and non-transplanted livers. 22

Figure S5. Frequency distribution of the highest recorded bile pH (note values >7.8 default to 7.8) 23

Figure S6: Frequency distribution of the lowest recorded bile glucose, comparing transplanted and non-transplanted livers. 24

Figure S7: Frequency distribution of the largest difference between perfusate minus bile glucose concentrations (in mmol/L), comparing transplanted and non-transplanted livers. 24

Figure S8 (a to q): shows the scatter plots and Spearman correlation coefficients for machine perfusion variables with the MEAF score. 25

SDC: Statistical methods

Descriptive analysis of variables was done using Prism version 7 (GraphPad software, La Jolla, CA, USA) and further descriptive analysis and linear regression models were carried out in SAS version 7.13 (SAS Institute Inc., Cary, NC, USA). Distributions of continuous variables were compared between transplanted and non-transplanted livers using the Mann-Whitney U test, and Fisher's Exact Test was used for categorical variables. Survival analysis was performed using the Kaplan Meier method.

Linear regression models with MEAF scores as outcomes, regressed on the perfusion variables, were fitted after first normalising the outcome variables of interest. MEAF differed significantly from a normal distribution. The best fitting transformation for the MEAF score was a square root transformation. Univariable linear regression models were fitted with each of the perfusion variables in turn as the exposure variable. For perfusion variables that had skewed distributions and all positive values, the natural log-transform of the variable was also considered as a predictor and if this was more strongly associated with the viability score than the variable on the original scale then this association is reported instead. The perfusion variables that showed the strongest associations with the MEAF score in univariable models were then considered as candidate variables to build a multivariable model for the prediction of the MEAF score.

For the model selection process for the multivariable model, analysis was restricted to the patients who had complete data on all of the candidate variables to be considered for inclusion in the model (the complete case dataset). The distribution of the MEAF score was assessed for normality in the complete case dataset and it was found that a square-root transformation of the MEAF best fitted a Normal Distribution. Therefore, the square-root of the MEAF score was used as the outcome variable in the linear regression model, as it was in the exploratory analysis.

Univariable linear regression models with each of the candidate variables as covariates and the square-root of the MEAF score as the outcome variable were fitted in turn in the complete case dataset and the candidate variables were ranked in order of the strength of their association with the MEAF score. Each of the candidate variables were then added one at a time to a multivariable linear regression model, with the most strongly associated variables

from univariable models included first, and each time a variable was added it was retained in the model if it significantly improved the model fit (at the $p<0.1$ level). Any variables with effects becoming non-significant on adding new variables to the multivariable model were removed. The final model was arrived at once there was no further improvement in model fit from addition of any of the other candidate variables.

For the 42 cases which were excluded from the multivariable analysis because of missing values from the dataset, those missing variables were as follows:

ALT at 60 minutes n=9
ALT at 120 minutes n=15
Glucose rate of fall n=1
Lactate at 3 hours n=2
Lactate at 4 ours n=4
Bile volume at 2 hours n=3
Bile volume at 3 hours n=6
Bile volume at 4 hours n=1
Bile perfusate glucose peak difference n=1

There were no livers where there were missing data for bicarbonate administration, lactate at 1 and 2 hours, lowest bile glucose or peak bile pH.

Table S1: Reasons for discarding 49 livers that underwent NESLiP

**Reason for turning down liver**	**DBD**	**DCD**
ALT >6000	6	15
Bile chemistry		16
Bile chemistry and perfusate lactate		1
High perfusate lactate	1	
Poor perfusion on machine		2 (both steatotic)
Steatosis	1	
Failure to metabolise glucose	1	1
Fibrotic appearance		1
Donor history		1
Recipient death, no other centre accepted liver (blood group AB)	1	
Damage to bile duct		1
Clots in circuit from retrieval		1

Biopsies were not done in any of these livers to aid in decision making.

Table S2. Cause of death or graft loss and MEAF at transplant

Day post transplant	Graft loss or death	MEAF	Cause
0	Death		Intraoperative cardiac arrest secondary to pulmonary embolism. NESLiP begun while this was being managed
0	Death		Cardiac arrest during hepatectomy with massive blood loss
0	Death		Complications during hepatectomy
2	Death		Cardiac arrest on background of severe pulmonary hypertension
2	Graft loss		Primary non function
3	Graft loss	5.7	Steatotic liver with massive subcapsular haemorrhage on reperfusion requiring packing with consequent ischaemia
5	Graft loss	4.4	Hepatic artery thrombosis
9	Graft loss	2.4	Fulminant antibody mediated rejection
20	Graft loss	5.4	Hepatic artery thrombosis
32	Death	4.9	Multi-organ failure with sepsis
45	Death	5.6	Multi-organ failure and venous outflow stenosis
57	Death	3.2	Multi-organ failure and venous outflow stenosis
95	Death	8.1	Multi-organ failure
153	Death	1.6	Chronic rejection
229	Graft loss	7.9	Cholangiopathy; died day 306 from sepsis
360	Graft loss	4.2	Cholangiopathy secondary to hepatic artery thrombosis

Note: No MEAF available for first 3 cases as did not survive 3 days.

Table S3. Number of livers perfused and transplanted, or not transplanted, with variables previously reported by us to be associated with a successful transplant

Hepatocyte variables				Cholangiocyte variables			Number of livers			
Alanine transaminase <6000iu/L at 2 hours	Rate of lactate fall >4mmol/L/h/kg	Glucose concentration fall	Sodium bicarbonate use ≤30mmol/L	Peak bile pH >7.5	Bile glucose ≤3 mmol/L	Perfusate-bile concentration ≥10mmol/L	Livers donated after donor brain death		Livers donated after donor circulatory death	
							Used	Not used	Used	Not used
✓	✓	✓	✓	✓	✓	✓	31	1	23	1
✓	✓	✓	✓	✓	✓	✗	4		16	1
✓	✓	✓	✓	✓	✗	✓	3		3	
✓	✓	✓	✓	✓	✗	✗	2		7	2
✓	✓	✓	✓	o	o	o			1	1
✓	✓	✓	✓	✗	✓	✗			1	
✓	✓	✓	✓	✗	✗	✗	1			1
✓	✓	✓	✗	✓	✓	✓	6		13	
✓	✓	✓	✗	✓	✓	✗	1		3	1
✓	✓	✓	✗	✓	✗	✗				8
✓	✓	✓	✗	✗	✗	✗				1
✓	✓	o	✓	✓	✓	✗			1	
✓	✓	o	✓	✓	✗	✗		1		1
✓	✓	o	✗	o	o	o	1	1		
✓	✓	✗	✓	✓	✓	✓	1			
✓	✓	✗	✓	✓	✗	✓		1		
✓	o	✓	✓	✓	✓	✓	8		3	
✓	o	✓	✓	✓	✓	o			1	
✓	o	✓	✓	✓	✓	✗	4		6	
✓	o	✓	✓	✓	o	✗	1			
✓	o	✓	✓	✓	✗	✓	2			
✓	o	✓	✓	✓	✗	✗	1		1	4
✓	o	✓	✓	o	o	o	2			
✓	o	✓	✗	✓	✓	✗			1	
✓	o	✓	✗	✓	✗	✗				1
✓	o	o	✓	✓	✓	✗				1
✓	o	o	✓	o	o	o				1
✓	✗	✓	✓	✓	✗	✓	1			
✓	✗	✓	✗	✓	✓	✓			1	
✓	✗	✓	✗	✓	✗	✗				2
✗	✓	✓	✓	✓	✗	✓		1		1

✗	✓	✓	✓	✓	✗	✗				2
✗	✓	✓	✗	✓	✓	✓			1	
✗	✓	✓	✗	✓	✗	✓		2		
✗	✓	✓	✗	✓	✗	✗				1
✗	o	✓	✓	✓	✓	✓			2	
✗	o	✓	✓	o	o	o				1
✗	o	✓	✓	✗	✓	✗				1
✗	o	✓	✓	✗	✗	✗				1
✗	o	✓	✗	✓	✗	✓	1			
✗	o	✗	✓	✓	✗	✗				1
✗	✗	✓	✓	✓	✗	✗				1
✗	✗	✓	✗	✓	✗	✗				1
✗	✗	✓	✗	o	o	o		1		1
✗	✗	o	✓	✓	✓	✓				1
✗	✗	✗	✗	✓	o	✓				1
✗	✗	✗	✗	o	o	o		2		

✗: criterion not met; ✓: criterion met; o: no value recorded, either because the liver had not been weighed (for the lactate) or no bile was produced (for the bile parameters). Livers in the first row met all our previously published criteria; those in the second row met them all, even though only one of the two bile glucose conditions was met. Note weight-adjusted lactate was not met if the liver was not weighed. The other livers not meeting the initial criteria, but where a large proportion of livers were transplanted, were perfusions requiring >30mls supplementary bicarbonate.

Table S4 Distributions of liver donor and perfusion parameters in transplanted livers (n=154) and non-transplanted livers (n=49)

Variable	Transplanted livers (n=154)			Non-Transplanted livers (n=49)			Kruskal-Wallis p-value for difference
	N (%) Observed	Median (IQR)	Range	N (%) Observed	Median (IQR)	Range	
Additional bicarb 0-2 hours	154 (100)	20 (10, 30)	0, 60	49 (100)	30 (20, 40)	0, 70	<0.001
Additional bicarb 0-4 hours	154 (100)	20 (10, 30)	0, 60	48 (98)	30 (20, 45)	0, 70	<0.001
Additional bicarb 2-4 hours	154 (100)	0 (0, 0)	0, 15	49 (100)	0 (0, 0)	0, 10	<0.055
Withdrawal period	153 (99.4)	5 (0, 13)	0, 181	49 (100)	10 (6, 18)	0, 64	<0.007
ALT at 1 hour	145 (94.2)	1228 (643, 2036)	94, 8000	40 (81.6)	4240.5 (1856, 10873.5)	282, 28823	<0.001
Log(ALT at 1 hour)	145 (94.2)	7.11 (6.47, 7.62)	4.54, 8.99	40 (81.6)	8.35 (7.53, 9.29)	5.64, 10.27	<0.001
ALT at 2 hours	138 (89.6)	1484 (751, 2314)	111, 10439	36 (73.5)	4295.5 (2325, 6842)	328, 20599	<0.001
Log(ALT at 2 hours)	138 (89.6)	7.3 (6.62, 7.75)	4.71, 9.25	36 (73.5)	8.37 (7.75, 8.83)	5.79, 9.93	<0.001
Asystolic period	154 (100)	9 (0, 13)	0, 32	49 (100)	12 (9, 14)	0, 22	0.002
Bile - perfusate glucose peak difference	148 (96.1)	11.8 (8.68, 16.5)	-7.6, 29.02	42 (85.7)	5.85 (4, 10.1)	-1.6, 26.8	<0.001
Bile glucose lowest	147 (95.5)	1.1 (0.9, 2.3)	0.1, 17.8	41 (83.7)	6.9 (3, 12.3)	0.5, 25	<0.001
Bile peak pH	149 (96.8)	7.8 (7.69, 7.8)	7.38, 7.8	42 (85.7)	7.71 (7.61, 7.8)	7.14, 7.8	0.018
Bile volume at 2 hours	140 (90.9)	6.45 (0.2, 11.8)	0, 42.4	47 (95.9)	2 (0, 7.5)	0, 20.9	0.023
Bile volume at 3 hours	127 (82.5)	14.9 (4.4, 22.6)	0, 69.9	40 (81.6)	5.65 (0.5, 13.05)	0, 31.4	0.002
Bile volume at 4 hours	130 (84.4)	20.35 (9, 31)	0, 88.5	36 (73.5)	11 (5.6, 19.1)	0, 46.4	0.017
Cold ischaemia time	154 (100)	406 (345, 497)	91, 1001	49 (100)	444 (362, 492)	121, 719	0.400
Donor age	154 (100)	48 (30, 57)	10, 78	49 (100)	52.4 (38.1, 59)	18.7, 69.5	0.210
EsLiP duration	154 (100)	483.5 (389, 585)	123, 1238	48 (98)	351.5 (256, 445)	121, 3644	<0.001
Glucose rate of fall	150 (97.4)	3.68 (2.51, 5)	-4.93, 22.57	45 (91.8)	2.66 (1.12, 3.87)	-3.21, 12.6	<0.001
Lactate at 1 hour	152 (98.7)	1 (0.4, 2.8)	0.1, 12.5	47 (95.9)	5.4 (1.2, 8.3)	0.1, 18.1	<0.001
Lactate at 2 hours	150 (97.4)	0.6 (0.3, 1.3)	0.1, 4.5	47 (95.9)	1 (0.6, 2.5)	0.1, 12.3	0.005
Lactate at 3 hours	147 (95.5)	0.7 (0.4, 1.3)	0.1, 16	45 (91.8)	1.1 (0.6, 2.4)	0.1, 11.3	0.010
Lactate at 4 hours	144 (93.5)	0.6 (0.3, 1.2)	0.1, 17	39 (79.6)	0.9 (0.5, 2.2)	0.1, 4.5	0.009
Lactate Peak rate fall in first hour	153 (99.4)	17.5 (12.9, 22.4)	3.4, 55.3	49 (100)	12 (8.5, 16.5)	2.6, 23	<0.001

Variable	Transplanted livers (n=154)			Non-Transplanted livers (n=49)			Kruskal-Wallis p-value for difference
	N (%) Observed	Median (IQR)	Range	N (%) Observed	Median (IQR)	Range	
Log (Lactate Peak rate fall in first hour)	153 (99.4)	2.86 (2.56, 3.11)	1.22, 4.01	49 (100)	2.48 (2.14, 2.8)	0.96, 3.14	<0.001
Liver weight	122 (79.2)	1543 (1332, 1800)	614, 2800	37 (75.5)	1755 (1535, 1982)	1016, 2240	0.020
UKDLI	154 (100)	1.43 (1.05, 1.86)	0.64, 2.79	49 (100)	1.84 (1.44, 2.13)	0.95, 2.6	<0.001
Feng DRI	154 (100)	2.03 (1.66, 2.44)	1.06, 3.57	3 (6.1)	2.72 (2.41, 3.15)	2.41, 3.15	0.033

Table S5. Spearman correlation coefficients of machine perfusion variables versus the MEAF Score

Variable	MEAF score reported (N=150)	
	**N (%) Observed**	**Spearman correlation coefficient with MEAF**
**Machine perfusion variables**		
ALT at 1 hour	141 (94)	0.40962
ALT at 2 hours	135 (90)	0.42203
Glucose rate of fall	146 (97.3)	-0.12901
Additional bicarb 0-2 hours	150 (100)	0.02028
Additional bicarb 0-4 hours	150 (100)	0.04331
Additional bicarb 2-4 hours	150 (100)	-0.01336
Lactate at 1 hour	148 (98.7)	0.19911
Lactate at 2 hours	146 (97.3)	0.26093
Lactate at 3 hours	143 (95.3)	0.27738
Lactate at 4 hours	141 (94)	0.26324
Lactate Peak rate fall in first hour	149 (99.3)	-0.05612
Bile volume at 2 hours	136 (90.7)	-0.23362
Bile volume at 3 hours	123 (82)	-0.24568
Bile volume at 4 hours	126 (84)	-0.27714
Bile perfusate glucose peak difference	144 (96)	-0.07523
Bile glucose lowest	144 (96)	0.27303
Bile peak pH	145 (96.7)	0.13184
**Other continuous variables**		
Agonal phase	149 (99.3)	-0.00978
Asystolic time	150 (100)	0.03252
Cold ischaemia time	150 (100)	0.08747
Donor age	150 (100)	0.21948
EsLiP duration	150 (100)	0.02835
Liver weight	120 (80)	0.23709
UKDLI	150 (100)	0.06020
Feng DRI	150 (100)	0.11900
creatinine ratio of peak to baseline	149 (99.3)	0.28522
ESLiP to implant	150 (100)	0.12763
UKELD	150 (100)	0.05098
MELD	150 (100)	-0.03457
MELD Na	150 (100)	0.03470
Preservation time	150 (100)	0.02964
Recipient age	150 (100)	-0.04045

Table S6. Mean difference in square-root-transformed MEAF score associated with the non-perfusion related variables included in the dataset in univariable linear regression models

Variable	Level	N	Mean diff	95% confidence interval	P-value	Overall p-value
Agonal phase	Linear	149	0.000015	(-0.0041, 0.0042)	0.990	0.990
Asystolic time	Linear	150	0.0030	(-0.0084, 0.015)	0.600	0.600
Cold ischaemia time	Linear	150	0.00026	(-0.00037, 0.0009)	0.420	0.420
Donor age	Linear	150	0.0077	(0.0026, 0.013)	0.003	0.003
EsLiP duration	Linear	150	-0.000082	(-0.00056, 0.00039)	0.730	0.730
Liver weight	Linear	120	0.00038	(0.00014, 0.00062)	0.002	0.002
UK DLI	Linear	150	0.035	(-0.12, 0.20)	0.660	0.660
US DRI	Linear	150	0.088	(-0.073, 0.25)	0.280	0.280
creatinine ratio of peak to baseline	Linear	149	0.18	(0.081, 0.27)	<0.001	<0.001
ESLiP to implant	Linear	150	0.0038	(-0.00072, 0.0083)	0.098	0.098
UKELD	Linear	150	0.0041	(-0.011, 0.019)	0.590	0.590
MELD	Linear	150	0.0074	(-0.0062, 0.021)	0.280	0.280
MELD Na	Linear	150	0.0037	(-0.0089, 0.016)	0.560	0.560
Preservation time	Linear	150	0.000073	(-0.00032, 0.00047)	0.720	0.720
Recipient age	Linear	150	-0.0034	(-0.011, 0.0038)	0.350	0.350
Donor type	DBD	68	0	-	-	0.770
	DCD	82	-0.025	(-0.20, 0.15)	0.770	
NRP utilised?	No	133	0	-	-	0.016
	Yes	17	0.32	(0.06, 0.59)	0.016	
Super-urgent patient	No	143	0	-	-	0.840
	Yes	7	-0.040	(-0.44, 0.36)	0.840	
Liver graft number	1	138	0	-	-	0.810
	2	10	0.090	(-0.25, 0.43)	0.600	
	3	1	0.17	(-0.88, 1.22)	0.750	
	4	1	-0.40	(-1.45, 0.65)	0.450	
Liver transplant	First	138	0	-	-	0.730
	Retx	12	0.056	(-0.26, 0.37)	0.730	

Table S7. Distributions of candidate machine perfusion variables and the MEAF score in the full dataset (n= 150) and for livers with complete data on all candidate variables as well as the MEAF (n = 108)

Variable		Full dataset (n=150)		Complete Case (n=108)
		N (%) Observed	Median (IQR) or N(%)	Median (IQR) or N (%)
MEAF Score	Linear	150 (100)	3.8 (2.7, 5.4)	3.7 (2.55, 5.4)
	Logged	150 (100)	1.34 (0.99, 1.69)	1.31 (0.94, 1.69)
	Square root	150 (100)	1.95 (1.64, 2.32)	1.92 (1.6, 2.32)
ALT at 1 hour	Linear	141 (94)	1206 (612, 1982)	1217 (595, 1978)
	Logged	141 (94)	7.1 (6.42, 7.59)	7.1 (6.39, 7.59)
ALT at 2 hours	Linear	135 (90)	1459 (695, 2314)	1352.5 (681.5, 2150)
	Logged	135 (90)	7.29 (6.54, 7.75)	7.21 (6.52, 7.67)
Glucose rate of fall	Linear	146 (97)	3.64 (2.51, 5)	3.99 (2.76, 5.17)
Additional bicarb 0-2 hours	Linear	150 (100)	20 (10, 30)	20 (12.5, 30)
Additional bicarb 0-2 hours (proportion of cohort)	0-10		46 (30.7)	27 (25)
	11-29		59 (39.3)	41 (38)
	>=30		45 (30)	40 (37)
Additional bicarb 0-4 hours	Linear	150 (100)	20 (10, 30)	20 (12.5, 30)
Additional bicarb 0-4 hours (proportion of cohort)	0-10		44 (29.3)	27 (25)
	11-29		60 (40)	41 (38)
	>=30		46 (30.7)	40 (37)
Additional bicarb 2-4 hours	Linear	150 (100)	0 (0, 0)	0 (0, 0)
Additional bicarb 2-4 hours (proportion of cohort)	0		145 (96.7)	104 (96.3)
	>0		5 (3.3)	4 (3.7)
Lactate at 1 hour	Linear	148 (99)	0.95 (0.4, 2.7)	1.15 (0.4, 2.8)
Lactate at 1 hour <1 (proportion of cohort)	No		74 (49.3)	57 (52.8)
	Yes		74 (49.3)	51 (47.2)
	Unknown		2 (1.3)	0
Lactate at 2 hours	Linear	146 (97)	0.6 (0.3, 1.3)	0.6 (0.2, 1.35)
Lactate at 2 hour <1 (proportion of cohort)	No		50 (33.3)	36 (33.3)
	Yes		96 (64)	72 (66.7)
	Unknown		4 (2.7)	0
Lactate at 3 hours	Linear	143 (95)	0.7 (0.3, 1.3)	0.6 (0.3, 1.2)
Lactate at 3 hour <1 (proportion of cohort)	No		49 (32.7)	34 (31.5)
	Yes		94 (62.7)	74 (68.5)
	Unknown		7 (4.7)	0
Lactate at 4 hours	Linear	141 (94)	0.6 (0.3, 1.2)	0.6 (0.3, 1.1)
Lactate at 4 hour <1 (proportion of cohort)	No		47 (31.3)	35 (32.4)
	Yes		94 (62.7)	73 (67.6)
	Unknown		9 (6)	0
Lactate Peak rate fall in first hour	Linear	149 (99)	17.6 (13.1, 22.5)	18 (13.65, 22.75)
	Logged	149 (99)	2.87 (2.57, 3.11)	2.89 (2.61, 3.12)
Bile volume at 2 hours	Linear	136 (91)	5.85 (0.2, 11.8)	7.25 (1.35, 12.85)
Bile volume at 3 hours	Linear	123 (82)	14.8 (4.4, 22.6)	15 (5.1, 23.2)
Bile volume at 4 hours	Linear	126 (84)	19.2 (7.95, 31)	24.05 (11.7, 31.85)
Bile perfusate glucose peak difference	Linear	144 (96)	11.8 (8.68, 16.5)	12.73 (9.85, 17.6)
Bile glucose lowest	Linear	144 (96)	1.1 (0.95, 2.2)	1.1 (0.9, 1.7)
Bile peak pH	Linear	145 (97)	7.79 (7.69, 7.8)	7.8 (7.71, 7.8)

Table S8. Distributions of other variables in full and complete candidate variable cohort

Variable		Full dataset (n=150)		Complete Case (n=108)	
		N (%) Observed	Median (IQR) or N(%)	N (%) Observed	Median (IQR) or N (%)
Agonal phase	Linear	149 (99)	5 (0, 13)	107 (99)	7 (0, 14)
Asystolic time	Linear	150 (100)	9 (0, 13)	108 (100)	9.5 (0, 13)
Cold ischaemia time	Linear	150 (100)	405.5 (343, 493)	108 (100)	411 (345, 489)
Donor age	Linear	150 (100)	48 (30, 56)	108 (100)	46.2 (29.95, 55.75)
EsLiP duration	Linear	150 (100)	479.5 (389, 582)	108 (100)	502 (421, 594.5)
Liver weight	Linear	120 (80)	1547 (1331, 1803.5)	97 (90)	1552 (1340, 1770)
UK DLI	Linear	150 (100)	1.43 (1.05, 1.85)	108 (100)	1.49 (1.04, 1.86)
US DRI	Linear	150 (100)	2.03 (1.66, 2.41)	108 (100)	2.03 (1.67, 2.38)
creatinine ratio of peak to baseline	Linear	149 (99)	1.5 (1.2, 2.1)	107 (99)	1.5 (1.2, 2.1)
ESLiP to implant	Linear	150 (100)	71 (59, 81)	108 (100)	73.5 (61, 84)
UKELD	Linear	150 (100)	53.9 (51.1, 58.4)	108 (100)	54.1 (51.2, 59.25)
MELD	Linear	150 (100)	13.9 (11.1, 18.5)	108 (100)	14 (11.4, 18.7)
MELD Na	Linear	150 (100)	16.9 (12.9, 21.5)	108 (100)	17.1 (12.85, 23)
Preservation time	Linear	150 (100)	965 (837, 1101)	108 (100)	1006 (899, 1139.5)
Recipient age	Linear	150 (100)	56 (48, 62)	108 (100)	55.85 (48.95, 62.5)
Donor type	DBD		68 (45.3)		47 (43.5)
	DCD		82 (54.7)		61 (56.5)
NRP utilised?	No		133 (88.7)		95 (88)
	Yes		17 (11.3)		13 (12)
Super-urgent patient	No		143 (95.3)		102 (94.4)
	Yes		7 (4.7)		6 (5.6)
Liver graft number	1		138 (92)		100 (92.6)
	2		10 (6.7)		7 (6.5)
	3		1 (0.7)		1 (0.9)
	4		1 (0.7)		0 (0)
Liver transplant	First		138 (92)		100 (92.6)
	Retx		12 (8)		8 (7.4)

Table S9. Mean difference in square-root transformed MEAF score associated with each of the machine perfusion variables in univariable linear regression models

Variable	Level	N	Mean diff	95% confidence interval	P-value	Overall p-value
ALT at 1 hour	Linear	141	0.00019	(0.00012, 0.00027)	<.001	<0.001
	Logged	141	0.27	(0.17, 0.37)	<.001	<0.001
ALT at 2 hours	Linear	135	0.00014	(0.000078, 0.00019)	<.001	<0.001
	Logged	135	0.26	(0.17, 0.36)	<.001	<0.001
Glucose rate of fall	Linear	146	-0.024	(-0.065, 0.017)	0.250	0.250
Additional bicarb 0-2 hours	Linear	150	0.0022	(-0.0041, 0.0084)	0.490	0.490
Additional bicarb 0-2 hours	0-10	46	0	-	-	0.400
	11-29	59	0.13	(-0.0716, 0.34)	0.200	
	>=30	45	0.034	(-0.18, 0.25)	0.760	
Additional bicarb 0-4 hours	Linear	150	0.0028	(-0.0032, 0.0089)	0.360	0.360
Additional bicarb 0-4 hours	0-10	44	0	-	-	0.020
	11-29	60	0.28	(0.073, 0.48)	0.008	
	>=30	45	0.093	(-0.12, 0.31)	0.390	
Additional bicarb 2-4 hours	Linear	150	-0.0087	(-0.051, 0.034)	0.680	0.680
Additional bicarb 2-4 hours	0	145	0	-	-	0.630
	>0	5	-0.12	(-0.59, 0.36)	0.630	
Lactate at 1 hour	Linear	148	0.039	(0.0046, 0.072)	0.026	0.026
Lactate at 1 hour <1	No	74	0	-	-	0.026
	Yes	74	-0.19	(-0.36, -0.024)	0.026	
Lactate at 2 hours	Linear	146	0.17	(0.069, 0.27)	0.001	0.001
Lactate at 2 hours <1	No	50	0	-	-	0.007
	Yes	96	-0.25	(-0.42, -0.067)	0.007	
Lactate at 3 hours	Linear	143	0.068	(0.0087, 0.13)	0.025	0.025
Lactate at 3 hours <1	No	49	0	-	-	0.008
	Yes	94	-0.24	(-0.42, -0.056)	0.011	0.011
Lactate at 4 hours	Linear	141	0.030	(-0.027, 0.087)	0.300	0.300
Lactate at 4 hours <1	No	47	0	-	-	0.005
	Yes	94	-0.25	(-0.44, -0.071)	0.007	0.007
Lactate at 4 hours <2	No	12	0			
	Yes	129	-0.23	(-0.54, 0.081)	0.140	0.140
Lactate at 4 hours <2.5	No	8	0			
	Yes	133	-0.16	(-0.54, 0.22)	0.410	0.410
Lactate Peak rate fall in first hour	Linear	149	-0.0055	(-0.016, 0.0055)	0.320	0.320
	Logged	149	-0.056	(-0.24, 0.13)	0.560	0.560
Bile volume at 2 hours	Linear	136	-0.013	(-0.023, -0.0034)	0.009	0.009
Bile volume at 3 hours	Linear	123	-0.0086	(-0.015, -0.0019)	0.012	0.012
Bile volume at 4 hours	Linear	126	-0.0064	(-0.011, -0.0015)	0.011	0.011
Bile perfusate glucose peak difference	Linear	144	-0.010	(-0.025, 0.0049)	0.180	0.180

Bile glucose lowest	Linear	144	0.032	(0.0025, 0.061)	0.033	0.033
Bile peak pH	Linear	145	0.73	(-0.25, 1.72)	0.140	0.140

Table S10. Mean difference in the square-root of the MEAF score associated with a unit increase in each of the machine perfusion variables in univariable linear regression model

Variable		Full dataset		Complete case cohort	
		Mean difference (95% CI)	Overall P-value	Mean difference (95% CI)	P-value
**Factors significant in univariable model**					
ALT at 1 hour	Logged	0.27 (0.17, 0.37)	<0.001	0.27 (0.16, 0.38)	<0.001
ALT at 2 hours	Logged	0.26 (0.17, 0.36)	<0.001	0.27 (0.16, 0.38)	<0.001
Glucose rate of fall	Linear	-0.024 (-0.065, 0.017)	0.250	-0.044 (-0.099, 0.010)	0.110
Additional bicarb 0-2 hours	Linear	0.0022 (-0.0041, 0.0084)	0.490	0.0016 (-0.0060, 0.0092)	0.670
Additional bicarb 0-2 hours	0-10	0	0.400	0	0.096
	11-29	0.13 (-0.0716, 0.34)	-	0.27 (0.0062,0.52)	-
	>=30	0.034 (-0.18, 0.25)	-	0.074 (-0.19, 0.33)	-
Additional bicarb 0-4 hours	Linear	0.0028 (-0.0032, 0.0089)	0.360	0.0024 (-0.0048, 0.0096)	0.520
Additional bicarb 0-4 hours	0-10	0	0.023	0	0.008
	11-29	0.28 (0.073, 0.48)	-	0.39 (0.14, 0.65)	-
	>=30	0.093 (-0.12, 0.31)	-	0.15 (-0.10, 0.41)	-
Additional bicarb 2-4 hours	Linear	-0.0087 (-0.051, 0.034)	0.680	0.0081 (-0.039, 0.055)	0.730
Additional bicarb 2-4 hours	0	0	0.630	0	0.730
	>0	-0.12 (-0.59, 0.36)	-	0.095 (-0.45, 0.64)	-
Lactate at 1 hour	Linear	0.039 (0.0046, 0.072)	0.026	0.042 (0.0020, 0.083)	0.040
Lactate at 1 hour <1	No	0	0.026	0 -	0.095
	Yes	-0.19 (-0.36, -0.024)	-	-0.17 (-0.37, 0.031)	
Lactate at 2 hours	Linear	0.17 (0.069, 0.27)	0.001	0.20 (0.084, 0.31)	<0.001
Lactate at 2 hour <1	No	0	0.007	0 -	0.007
	Yes	-0.25 (-0.42, -0.067)	-	-0.29 (-0.50, -0.083)	
Lactate at 3 hours	Linear	0.068 (0.0087, 0.13)	0.025	0.065 (0.0036, 0.13)	0.038
Lactate at 3 hour <1	No	0 -	0.011	0 -	0.016
	Yes	-0.24 (-0.42, -0.056)	-	-0.26 (-0.48, -0.049)	
Lactate at 4 hours	Linear	0.03 (-0.027, 0.087)	0.3	0.024 (-0.036, 0.083)	0.430
Lactate at 4 hour <1	No	0 -	0.007	0	0.030
	Yes	-0.25 (-0.44, -0.071)	-	-0.24 (-0.45, -0.024)	
Lactate at 4 hour <2	No	0	0.140	0	0.290
	Yes	-0.23 (-0.54, 0.081)	-	-0.20 (-0.57, 0.17)	-
Lactate at 4 hour <2.5	No	0	0.410	0	0.530
	Yes	-0.16 (-0.54, 0.22)	-	-0.14 (-0.59, 0.30)	-
Lactate Peak rate fall in first hour	Linear	-0.0055 (-0.016, 0.0055)	0.320	-0.0089 (-0.023, 0.0050)	0.210

	Logged	-0.056 (-0.24, 0.13)	0.560	-0.15 (-0.40, 0.10)	0.250
Bile volume at 2 hours	Linear	-0.013 (-0.023, -0.0034)	0.009	-0.0093 (-0.021, 0.0021)	0.110
Bile volume at 3 hours	Linear	-0.0086 (-0.015, -0.0019)	0.012	-0.0070 (-0.014, 0.00024)	0.058
Bile volume at 4 hours	Linear	-0.0064 (-0.011, -0.0015)	0.011	-0.0057 (-0.011, -0.00010)	0.046
Bile perfusate glucose peak difference	Linear	-0.01 (-0.025, 0.0049)	0.180	-0.0063 (-0.025, 0.013)	0.510
Bile glucose lowest	Linear	0.032 (0.0025, 0.061)	0.033	0.055 (-0.00006, 0.11)	0.050
Bile peak pH	Linear	0.73 (-0.25, 1.72)	0.140	1.09 (-0.086, 2.27)	0.069
Donor type	DBD	0	0.290	0	0.550
	DCD	-0.12 (-0.33, 0.10)	-	-0.063 (-0.27, 0.14)	-
Donor age	Linear	0.0077 (0.0026, 0.013)	0.004	0.0093 (0.0030, 0.016)	0.004
UKDLI	Linear	0.035 (-0.12, 0.20)	0.660	0.023 (-0.16, 0.21)	0.810
Feng DRI	Linear	0.088 (-0.073, 0.25)	0.280	0.070 (-0.12, 0.26)	0.470

Figure S1. Graft, patient and transplant survival

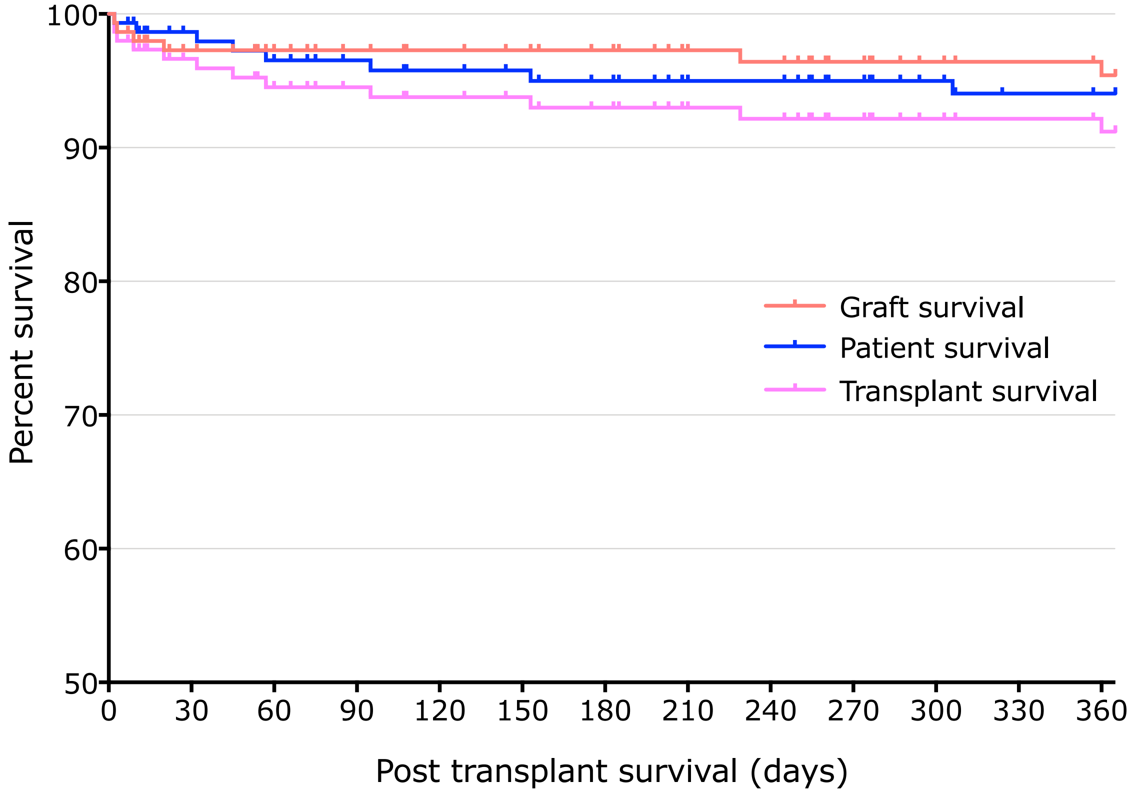

Graft survival is censored for patient death; transplant survival is graft survival not censored for patient death.

One year graft survival was 95.4%, patient survival 94%, and transplant survival 91.2%.

Figure S2a. Frequency distribution of the model for early allograft function (MEAF) scores for transplanted livers

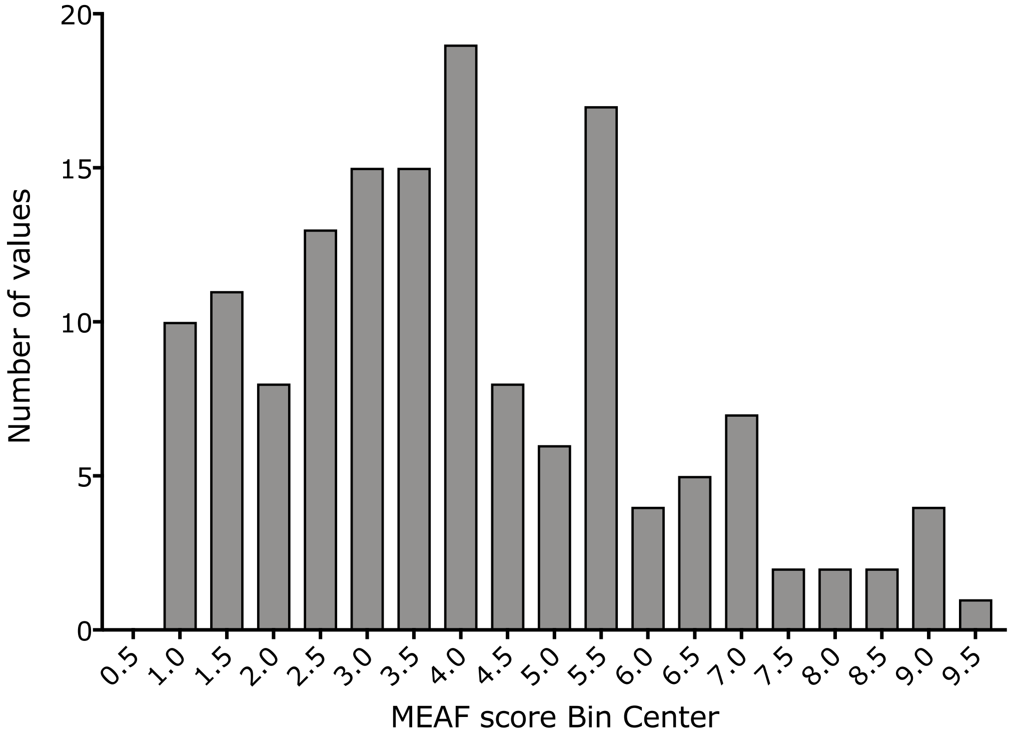

Figure S2b. Frequency distribution of the square root transformed Model for Early Allograft Function (MEAF) scores for transplanted livers

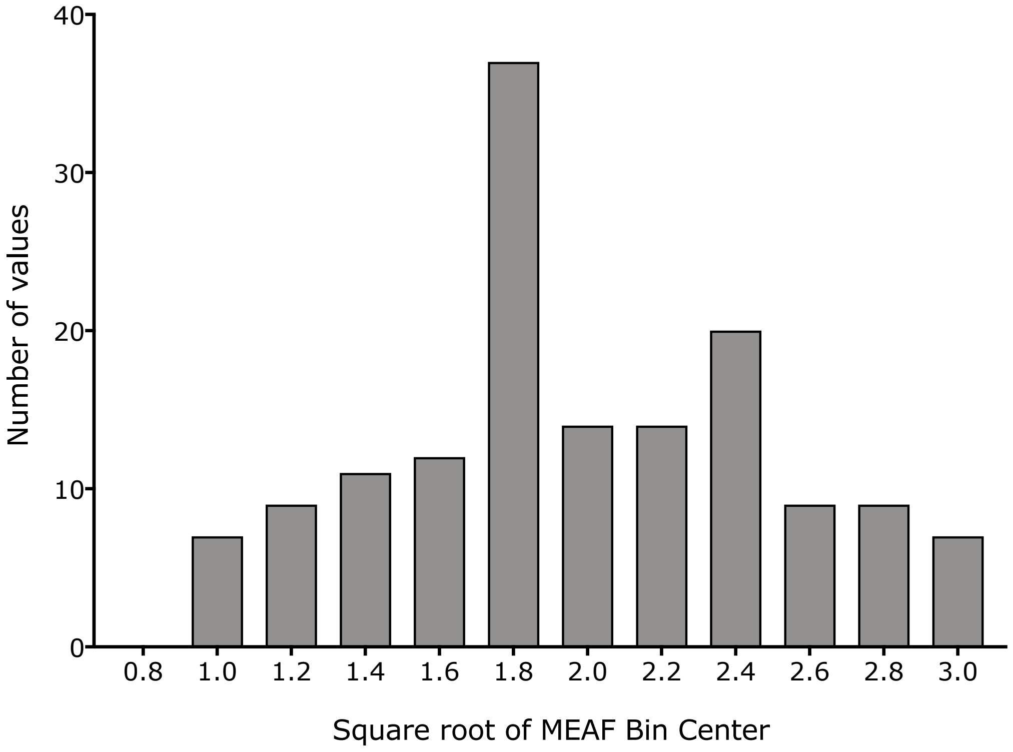

Figure S2a: Frequency distribution of 2-hour perfusate ALT concentrations in transplanted and non-transplanted livers

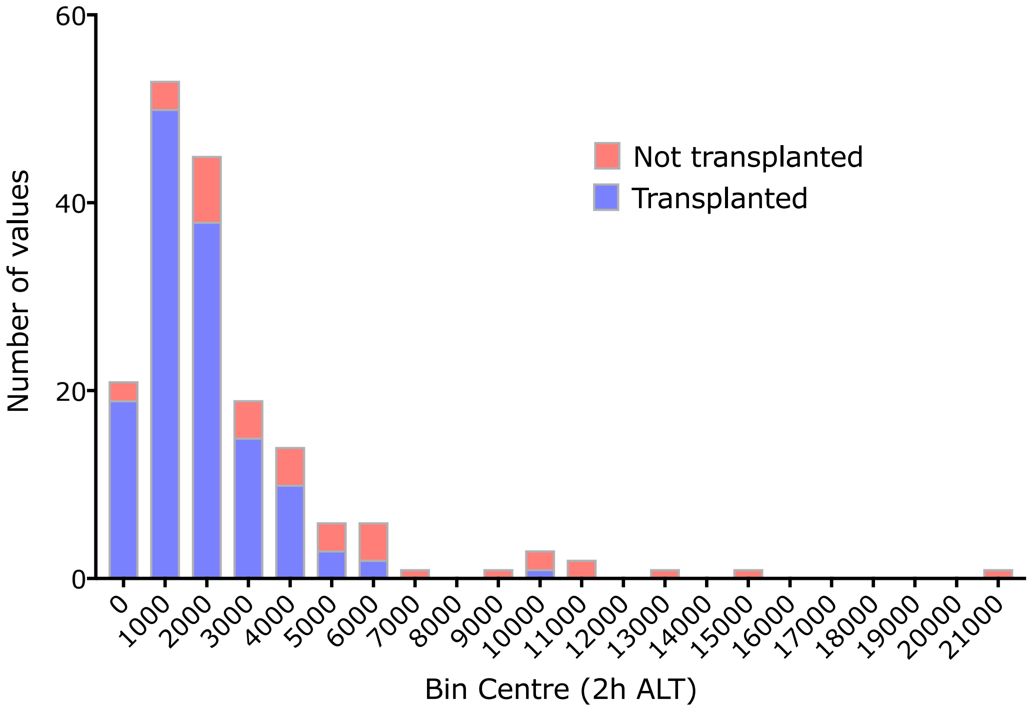

Figure S2b: Frequency distribution of the natural logarithm of the 2-hour perfusate ALT concentrations in transplanted and non-transplanted livers

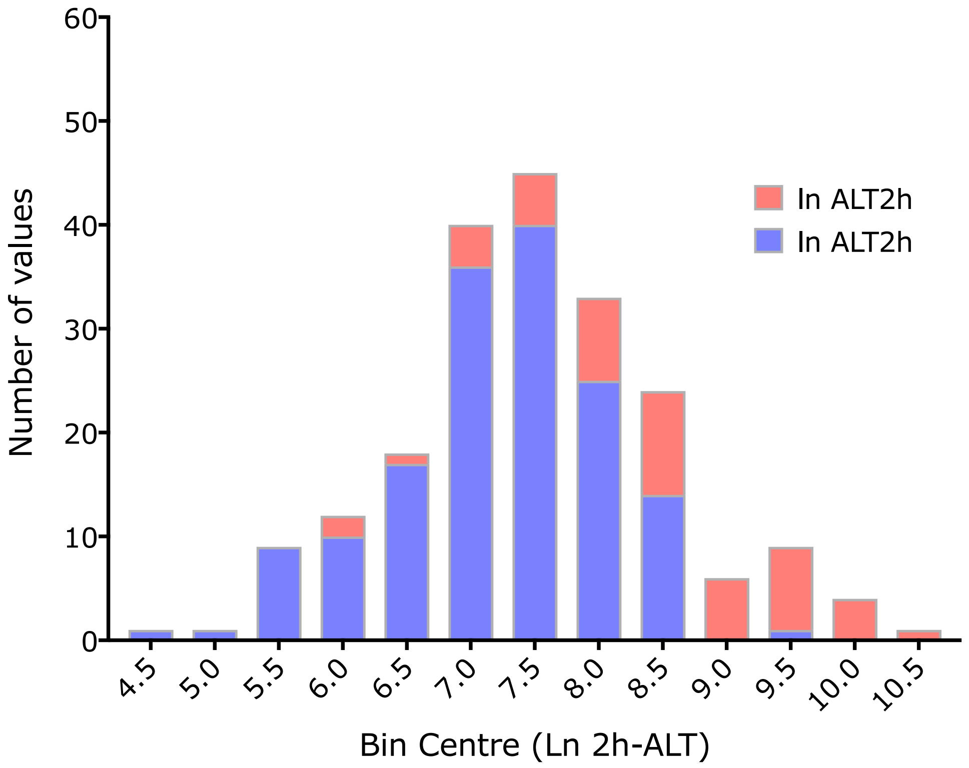

Figure S3. Frequency distribution of the 2-hour perfusate lactate concentration (mmol/L)

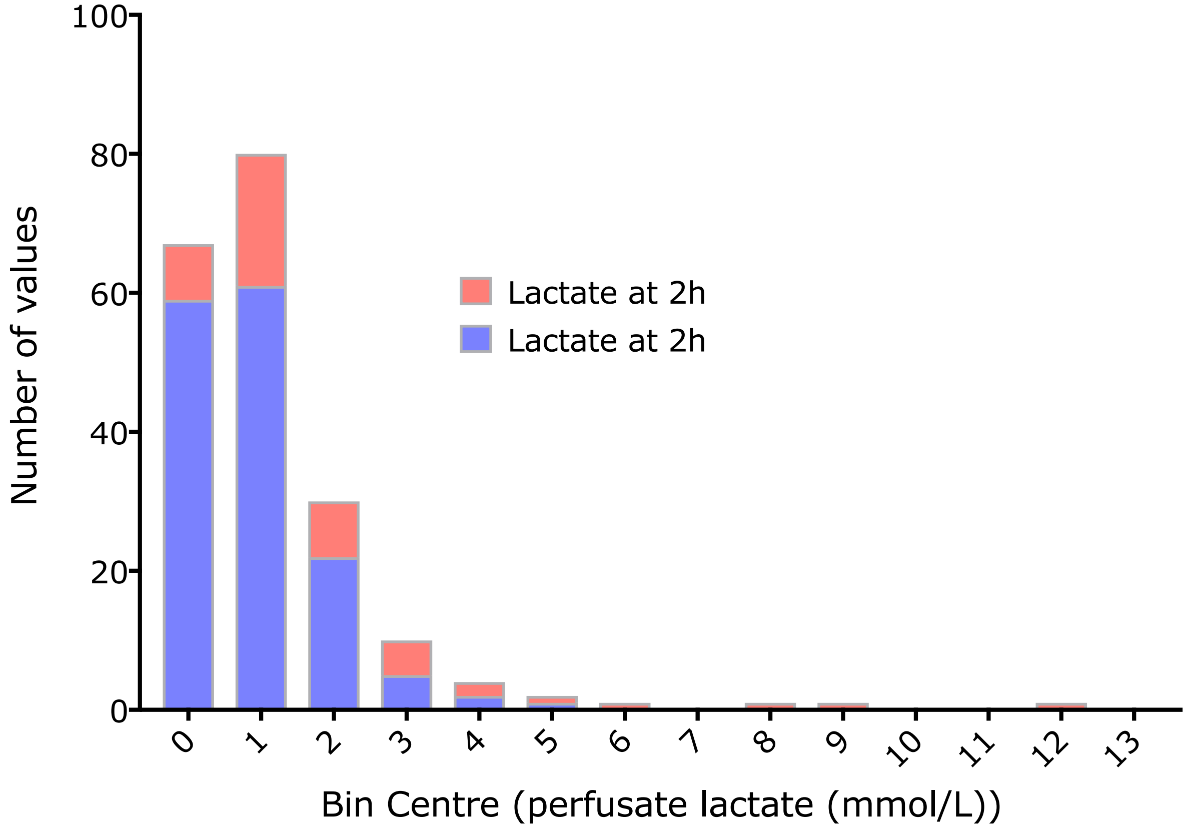

Figure S4. Frequency distribution of the number of millimoles of sodium bicarbonate supplementation required in the first 4 hours of perfusion to keep the perfusate pH >7.2, comparing transplanted and non-transplanted livers.

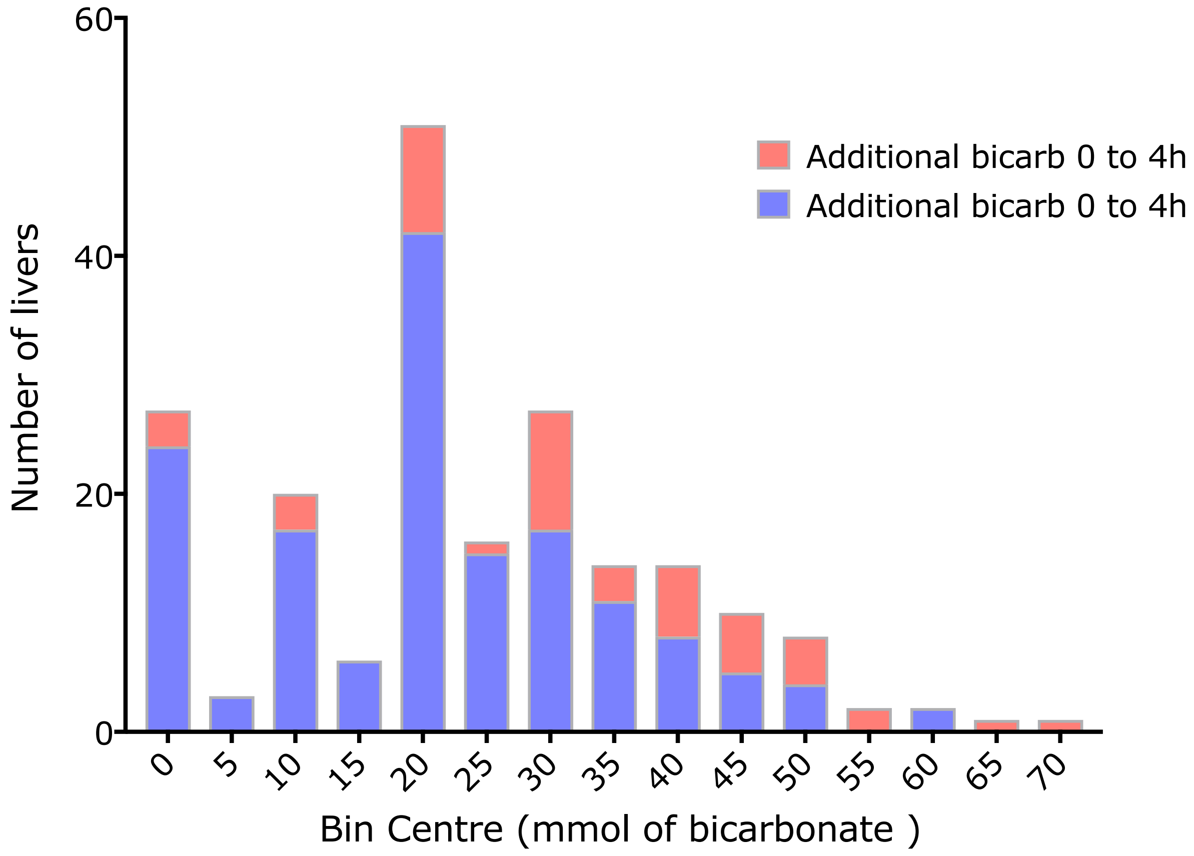

Figure S5. Frequency distribution of the highest recorded bile pH (note values >7.8 default to 7.8)

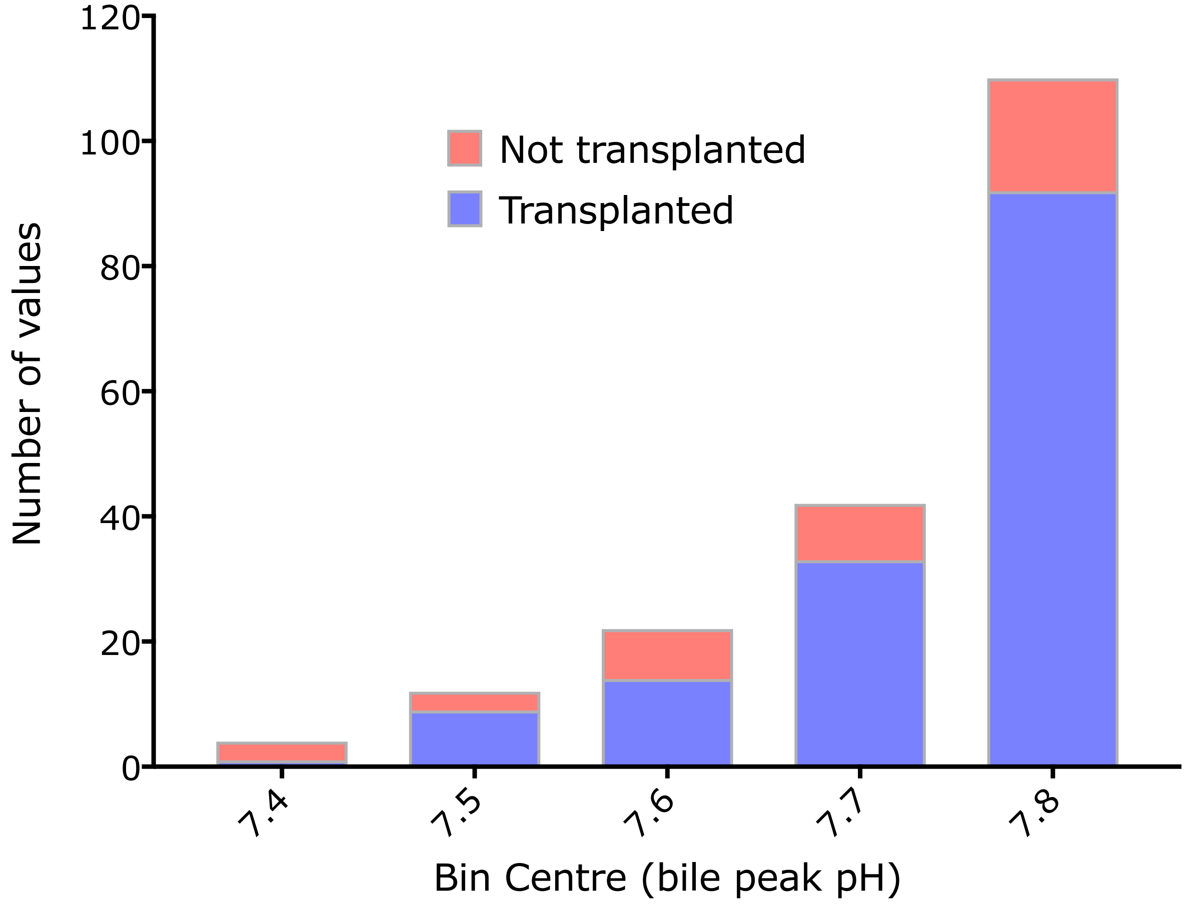

Figure S6: Frequency distribution of the lowest recorded bile glucose, comparing transplanted and non-transplanted livers.

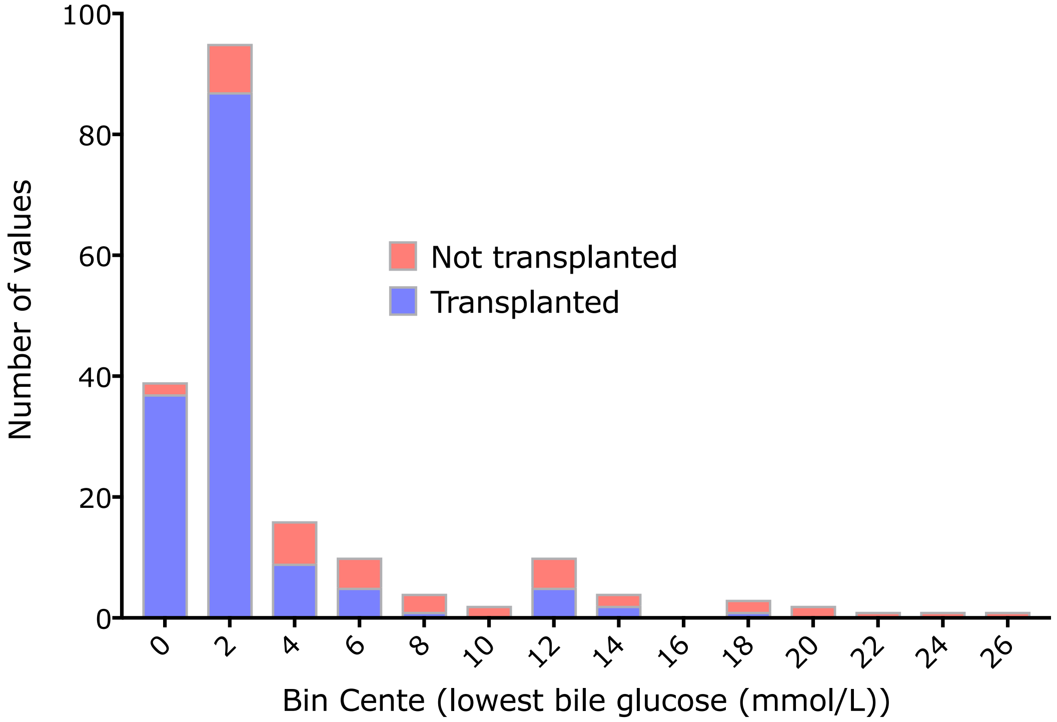

Figure S7: Frequency distribution of the largest difference between perfusate minus bile glucose concentrations (in mmol/L), comparing transplanted and non-transplanted livers.

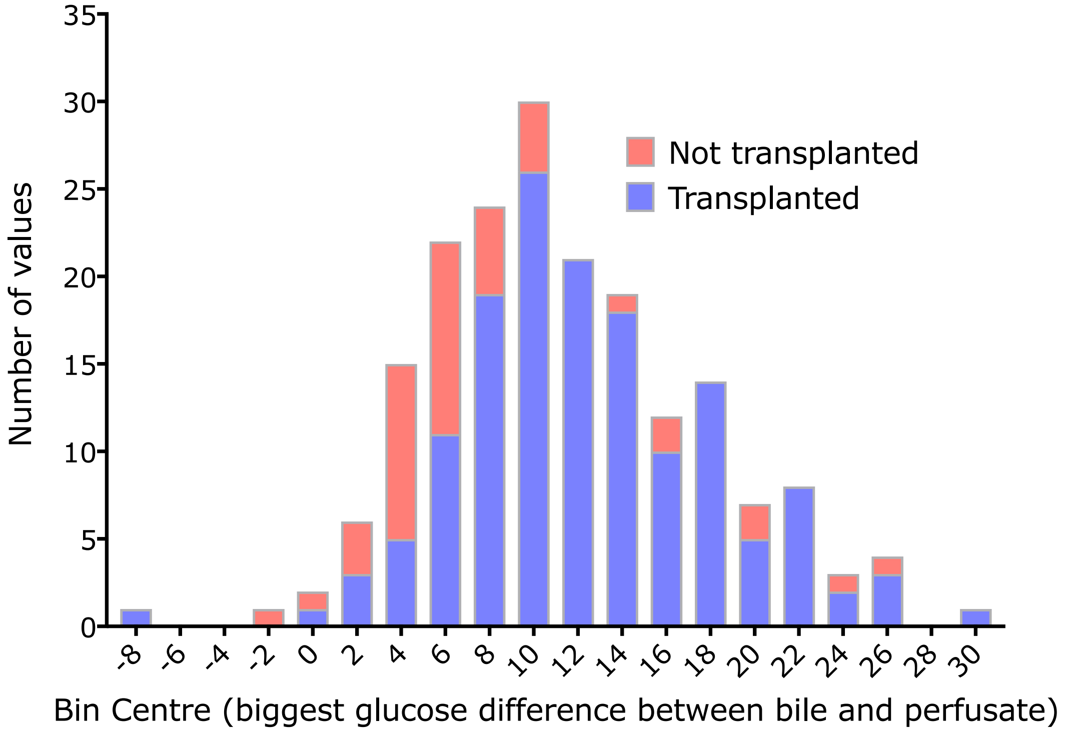

Figure S8 (a to q): shows the scatter plots and Spearman correlation coefficients for machine perfusion variables with the MEAF score.

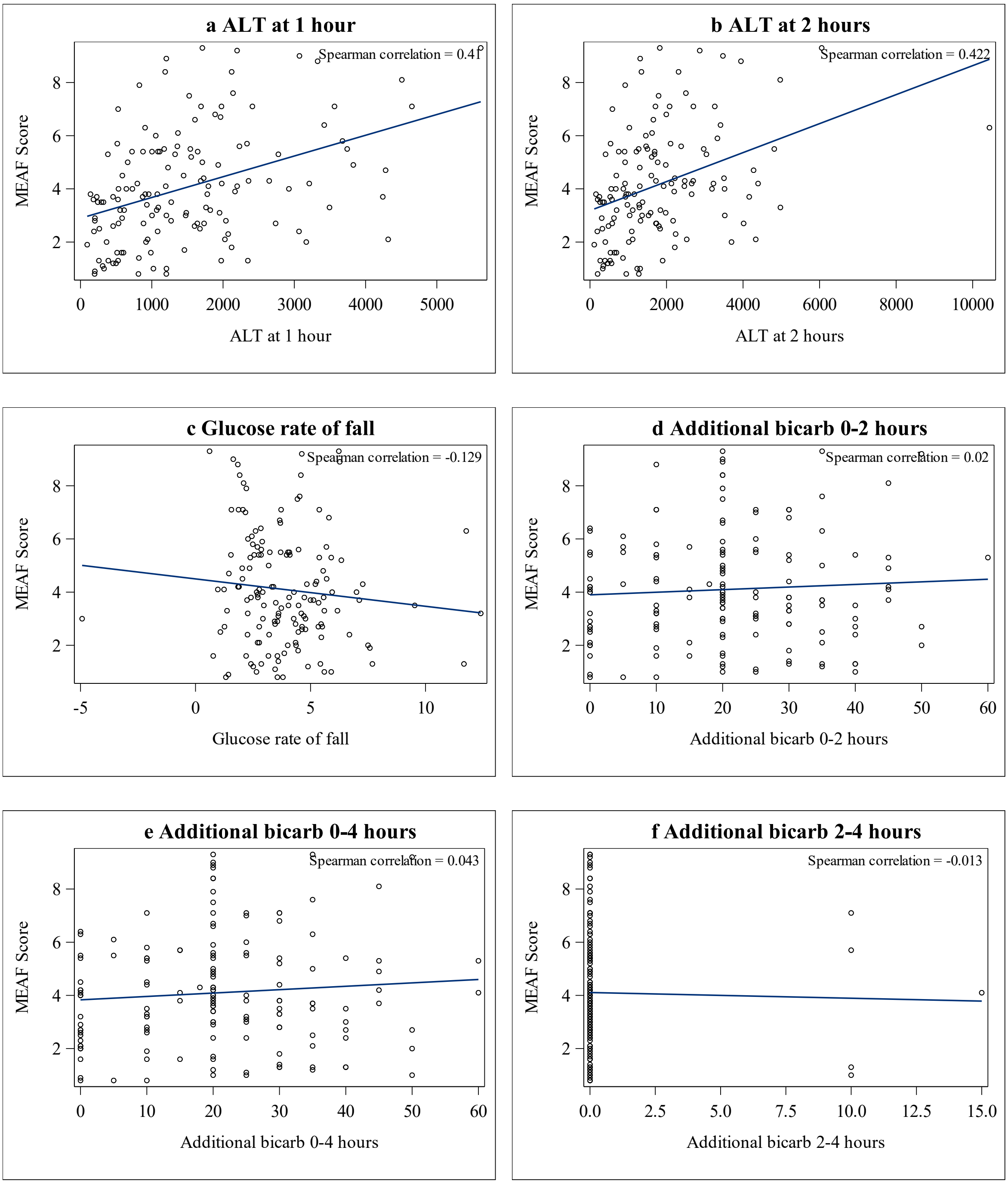

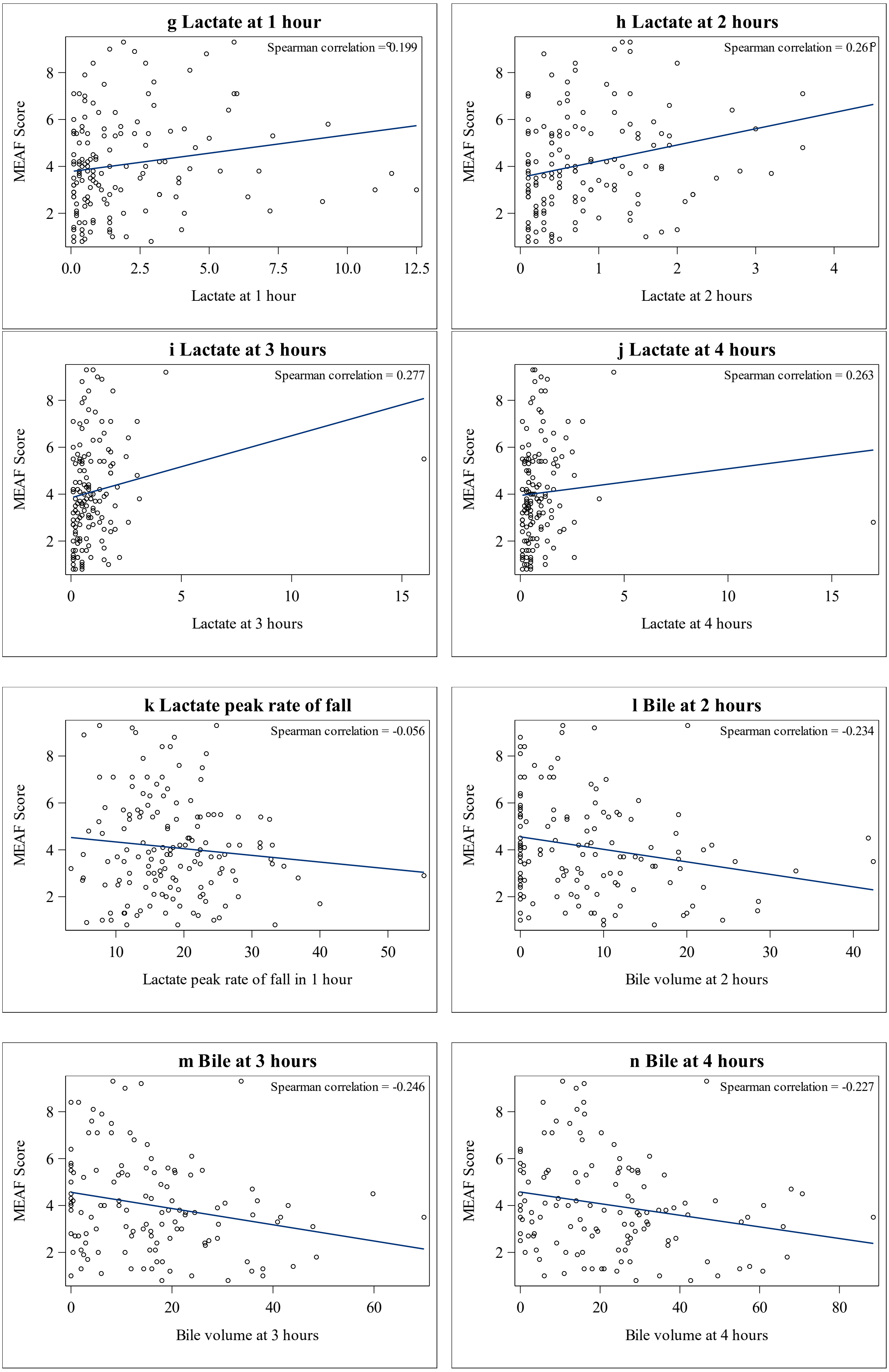
g Lactate at 1 hour
Spearman correlation = 0.199
MEAF Score
Lactate at 1 hour
h Lactate at 2 hours
Spearman correlation = 0.261
MEAF Score
Lactate at 2 hours
i Lactate at 3 hours
Spearman correlation = 0.277
MEAF Score
Lactate at 3 hours
j Lactate at 4 hours
Spearman correlation = 0.263
MEAF Score
Lactate at 4 hours
k Lactate peak rate of fall
Spearman correlation = -0.056
MEAF Score
Lactate peak rate of fall in 1 hour
l Bile at 2 hours
Spearman correlation = -0.234
MEAF Score
Bile volume at 2 hours
m Bile at 3 hours
Spearman correlation = -0.246
MEAF Score
Bile volume at 3 hours
n Bile at 4 hours
Spearman correlation = -0.227
MEAF Score
Bile volume at 4 hours

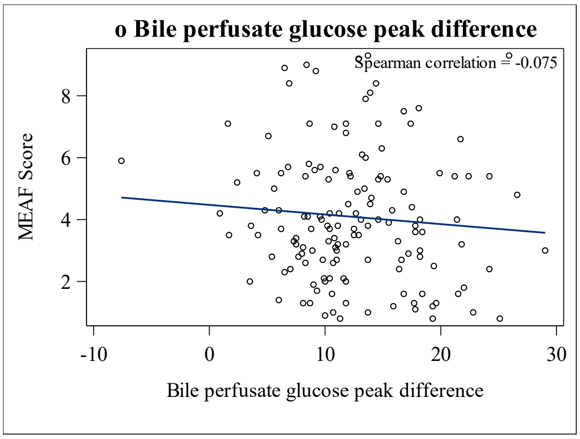
o Bile perfusate glucose peak difference
Spearman correlation = -0.075
MEAF Score
8
6
4
2
-10
0
10
20
30
Bile perfusate glucose peak difference

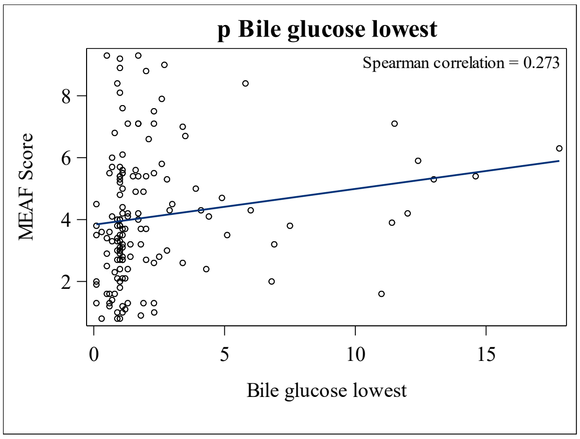
p Bile glucose lowest
Spearman correlation = 0.273
MEAF Score
8
6
4
2
0
5
10
15
Bile glucose lowest

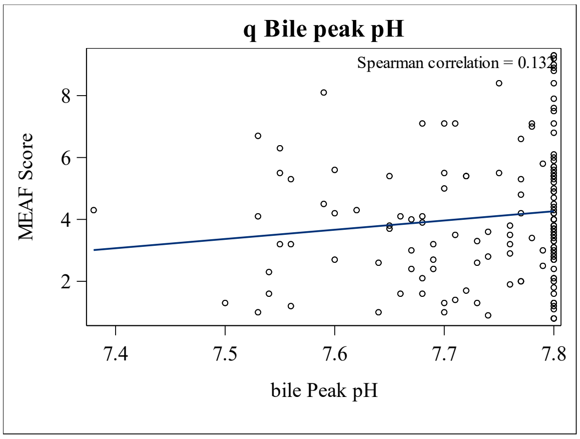
q Bile peak pH
Spearman correlation = 0.132
MEAF Score
8
6
4
2
7.4
7.5
7.6
7.7
7.8
bile Peak pH
